# CRISPR-Cas9 Genome and Double-Knockout Screening to Identify Novel Therapeutic Targets for Chemoresistance in Triple-Negative Breast Cancer

**DOI:** 10.3390/cancers17233876

**Published:** 2025-12-03

**Authors:** Shuai Shao, Shangjia Li, Yang Huo, Shan Tang, Birkan Gökbağ, Kunjie Fan, Yirui Huang, Lingling Wang, Gregory Nagy, Jeffrey Parvin, Daniel Stover, Lijun Cheng, Lang Li

**Affiliations:** 1 Department of Biomedical Informatics, College of Medicine, The Ohio State University, Columbus, OH 43210, USA; 2 College of Pharmacy, The Ohio State University, Columbus, OH 43210, USA; 3 Division of Medical Oncology, Department of Medicine, The Ohio State University, Columbus, OH 43210, USA

**Keywords:** triple-negative breast cancer, CRISPR-Cas9, genome screening

## Abstract

**Background/Objectives**: Triple-negative breast cancer (TNBC) accounts for 15 to 20% of breast cancer cases and contributes to a disproportionate 35% of breast cancer deaths. Its resistance to chemotherapy presents a significant challenge. **Methods**: We firstly compared transcriptomic profiles between TNBC cell lines and patient samples and inferred the MDA-MB-231 cell line as the most representative model for TNBC with poor response to chemotherapy. We then conducted a genome-wide CRISPR-Cas9 screening and RNA-seq analysis in MDA-MB-231. **Results**: This analysis revealed 96 and 93 genes that could re-sensitize cisplatin and doxorubicin treatment, respectively, with 19 overlapping genes. Among these genes, 28 have been studied and published previously in chemoresistance research. MCM9 was found as a new TNBC chemoresistance target. Its target drug, KPT-185, showed an additive effect with cisplatin in treating TNBC cells. In the follow-up gene combination double-knockout experiment among 65 genes selected from cell death pathways, 242 synthetic lethal gene pairs were discovered to overcome chemoresistance in TNBC. **Conclusions**: In this study, we identified synthetic lethal targets in treating TNBC with cisplatin and doxorubicin through a genome-wide CRISPR-Cas9 screening and gene combination double-knockout screening.

## Simple Summary

Triple-negative breast cancer (TNBC) accounts for 15 to 20% of breast cancer cases and contributes to a disproportionate 35% of breast cancer deaths. Its resistance to chemotherapy presents a significant challenge. In this study, we identified synthetic lethal targets in treating TNBC with cisplatin and doxorubicin through a genome-wide CRISPR-Cas9 screening.

## 1. Introduction

Triple-negative breast cancer (TNBC) is distinguished by the absence of the estrogen receptor (ER), progesterone receptor (PR), and human epidermal growth factor receptor 2 (HER2) [1], three pivotal receptors that substantially influence the proliferation and dissemination of breast cancer cells. In most breast cancer cases, therapeutic interventions target one or more of these receptors to impede or halt cancer progression, and their absence in TNBC presents a particularly formidable challenge to treatment [2].

The conventional treatment regimen for TNBC comprises a combination of surgical intervention, radiotherapy, and chemotherapy [3]. Cisplatin and doxorubicin, two front-line chemotherapeutic agents used to manage TNBC, both function to kill cancer cells by damaging their DNA. Platinum-based cisplatin does this by forming covalent bonds with the DNA [4], and doxorubicin, an anthracycline, encompasses DNA double helix intercalation, topoisomerase II inhibition, and free radical generation [5]. However, though these chemotherapeutic drugs can effectively eradicate tumor cells, patients with TNBC have demonstrated resistance to them [6,7]. Large clinical trials have shown residual cancer in approximately half of patients with TNBC following neoadjuvant chemotherapy (NACT) [8,9], and around 40% of those with residual disease will eventually develop distant metastasis [10]. Thus, it is important to identify more effective treatment strategies for patients with TNBC who respond poorly to these chemotherapeutic drugs.

In recent years, significant progress in understanding the molecular characteristics of TNBC has led to the development of potential new targeted and personalized therapeutic options for these patients, including the use of immunotherapies, poly (ADP-ribose) polymerase (PARP) inhibitors, antibody–drug conjugates (ADCs), and targeted therapies based on specific molecular mechanisms. Such immunotherapies as atezolizumab (Tecentriq^®^) [11] have shown encouraging results in combination with nab-paclitaxel in advanced triple-negative breast cancer, and PARP inhibitors like olaparib (Lynparza^®^) [12], niraparib (Zejula), and talazoparib (Talzenna^®^) [13] have shown promise, particularly when combined with such chemotherapeutic agents as paclitaxel, as first- or second-line treatment in patients with metastatic TNBC. ADCs [14,15], such as sacituzumab govitecan (Trodelvy^®^), selectively deliver cytotoxic agents to cancer cells while minimizing damage to healthy cells, and targeted therapies based on specific molecular mechanisms [16], like phosphoinositide 3-kinase (PI3K) inhibitors, e.g., alpelisib, buparlisib, and mammalian target of rapamycin (mTOR) inhibitors, e.g., everolimus, offer new avenues for TNBC treatment. The emerging cancer treatment capitalizes on the concept of synthetic lethality (SL), which occurs when the simultaneous inhibition or disruption of two genes results in cell death [17]. The SL between *BRAC* and *PARP* stems from the mechanism whereby it controls two complementary pathways in DNA homologous recombination repair [18]. Therefore, PARP inhibitors, olaparib and talazoparib, are promising for treating breast cancer patients with *BRCA1* or *BRCA2* mutations [19].

The CRISPR/Cas9 system is a powerful gene editing tool that disrupts or modifies gene functions [20]. Genome-wide CRISPR screening allows researchers to examine the entire genome systematically to identify genes whose knockout or modification can lead to particular phenotypes, such as increased sensitivity to chemotherapy or SL combined with another genetic disruption [21,22]. In this paper, for the first time, we conduct genome-wide CRISPR screening using the Toronto Knockout CRISPR library (TKOV3; Version 3) to identify potential druggable targets capable of overcoming the resistance in patients with TNBC to DNA-damaging chemotherapeutic agents, such as cisplatin and doxorubicin. Specifically, we investigate SL between new druggable targets and chemotherapeutic drugs that can overcome chemoresistance. We also draw genes from the XDeathDB database [23] to create a CRISPR double-knockout library [24] for cell death. Our strategy focuses on discovering SL gene pairs that could potentially serve as drug targets for TNBC.

Prior to initiating the CRISPR-Cas9 screening experiment, our selection of an appropriate and representative cell-line model was crucial. An improperly selected cell model that is not representative of TNBC-chemoresistant patients would prevent translational target and drug discoveries from preclinical to clinical research. Indeed, many drugs with promising preclinical results failed in clinical trials because the cell-line models do not accurately represent patients [25,26]. PubMed citations indicate that 40.2% of metastatic breast cancer research employs the MDA-MB-231 cell-line model. However, Chen’s research group [27] observed less resemblance of MDA-MB-231 to basal-like metastatic breast cancer patients. In a separate study of ovarian cancer, Domcke’s team [28] compared genomic profiles of 47 ovarian cancer cell lines with ovarian cancer tumor samples and found greater resemblance of several lesser-known cell lines with high-grade serous ovarian tumor samples than other widely used cell lines. Therefore, in this paper, proper TNBC chemoresistance cell models were selected before extensive target and drug discovery experiments using CRISPR-Cas9 screening technologies.

## 2. Materials and Methods

### 2.1. Triple Negative Breast Cancer Transcriptome Data Collection

Our reported query of the Gene Expression Omnibus (GEO) [29] using the keywords “breast cancer” and “chemo” to conduct pathway and sub-pathway analyses revealed the activation of several molecular pathways associated with chemoresistance in patients with breast cancer [29]. Tumor samples were categorized as either chemo-sensitive or -resistant according to the Miller–Payne (MP) five-grade system for assessing patient resistance to chemotherapy, which serves as the basis for determining chemoresistance in accordance with the standard cancer treatment protocol. In this paper, we focus on TNBC samples from three cohorts (Table 1). We also obtained genomic profiles of 21 TNBC cell lines from 916 cancer cells in the CCLE [30]. All samples were generated using the same Affymetrix Human Genome U133 Plus 2.0 Array platform (Affymetrix, Santa Clara, CA, USA).

### 2.2. Bioinformatics Data Analyses of Transcriptome Between TNBC Cell Lines and Tumor Samples

#### 2.2.1. Pre-Processing of Data for Gene Expression Profiles

We analyzed Affymetrix U133 Plus 2.0 microarray data after robust multiarray average (RMA) normalization using Expression Console™ (EC) software version 1.1 (Affymetrix). The signal intensity was logged with base 2 to stabilize variance. Probes were removed if expression levels in 80% of the samples were lower than the background noise. Affymetrix probe IDs were mapped to gene symbols based on their General Public License (GPL) platforms. When multiple probes mapped to a single gene, the median expression value was used in the analysis.

#### 2.2.2. Bach Effect Removal

Because our samples originated from various studies, to ensure an accurate and unbiased analysis, we employed the limma package in R [34] to correct for batch effects.

#### 2.2.3. Different Gene Expression and Hierarchical Cluster Analysis

We analyzed differential gene expression utilizing the limma package in R software (version 3.6.1) [35], adjusted nominal *p*-values using the Benjamini–Hochberg method, and calculated fold change by comparing mean expressions between non-response and response groups. We conducted multiple analyses for various purposes, including comparisons between non-response and response in baseline subjects (sample size: 29:15), non-response and response in post-chemotherapy subjects (sample size: 11:4), and post-chemotherapy and baseline conditions (sample size: 29:11). The hierarchical clustering analysis of TNBC clinical transcriptome data and CCLE cell line transcriptome data was performed in Heatmap in R.

#### 2.2.4. Correlation Analysis Between TNBC Cell Lines and TNBC Patient Samples Who Were Poor Responders to Chemotherapy

Affymetrix data in the CCLE dataset include over 10,000 genes across 916 cell lines. We first calculated the standard deviation of each gene across the 916 cell lines to ascertain variation in these genes in all CCLE cancer cells and selected the top 2000 variation genes for the follow-up analysis. We then calculated Spearman correlations in these 2000 genes between a given cell line and all the TNBC poor responders, with an average of all the correlations representative of the similarity between the cancer cell and poor responder population. A higher average correlation indicates better representation.

#### 2.2.5. ssGSEA Pathway Similarity Analysis

We obtained ssGSEA scores using R package gene set variation analysis (GSVA) [36] for the 50 MSigDB cancer hallmark gene sets [37]. Hierarchical clustering of ssGSEA scores were explored between TNBC cell lines and TNBC poor responder samples in the context of the cancer hallmark gene set.

#### 2.2.6. Gene Ontology (GO) and KEGG Pathway Analysis

GO [38] and Kyoto Encyclopedia of Genes and Genomes (KEGG) [39] gene enrichment analyses were used in identifying and interpreting complex molecular functions, biological processes, cellular components, and signaling pathways in drug resistance genes. These analyses were performed in clusterProfiler in R [40]. The threshold of statistically significance was a *p*-value below 0.05.

### 2.3. Cell Culture

Human TNBC cell lines MDA-MB-231, MDA-MB-436, and HS-578T, along with the human embryonic kidney (HEK) cell line 293T, were obtained from the American Type Culture Collection (Manassas, VA, USA) for this study. All cell lines were cultured in Ham’s F-12K (Kaighn’s) medium, supplemented with 10% fetal bovine serum (VWR, Radnor, PA, USA), 1% GlutMax, 1% sodium pyruvate, and penicillin–streptomycin (Gibco, Waltham, MA, USA). The cell lines were incubated at 37 °C in a 5% CO_2_ atmosphere. All cell lines underwent authentication via short tandem repeat (STR) profiling and were tested for mycoplasma contamination every three months.

#### 2.3.1. Cell Survival Assay Using siRNA-Mediated Gene Silencing

We used small interfering RNAs purchased from Thermo Fisher Scientific (Waltham, MA, USA) that were specifically designed to target and validate the essential genes identified in our study. Information regarding the siRNAs is detailed in the Supplementary SI Gene list. We transfected MDA-MB-231, MDA-MB-436, and HS578T cells with siRNAs using the Lipofectamine™ RNAiMAX transfection reagent kit (#13778150, Thermo Fisher Scientific) according to the manufacturer’s protocol. The cells were then seeded into 96-well plates at a density of 2.5 × 10^3^ cells per well. After 24 h, the medium was replaced, and the cells were treated with cisplatin and doxorubicin. Following 120 h of incubation, we assessed cell viability using the alamarBlue™ HS cell viability reagent (#A50100, Thermo Fisher Scientific). The absorbance of each well was measured using a microplate reader. We determined the half-maximal inhibitory concentration (IC50) values using GraphPad Prism 7 software.

#### 2.3.2. Cell Survival Assay Using Single Drug

MDA-MB-231 TNBC cells were seeded in 96-well plates at a density of 5000 cells per well in 90 µL medium and allowed to grow overnight. Subsequently, the cells were treated with chemotherapy drugs or molecular inhibitors in 10 µL medium (with 10% DMSO in it).

To comprehensively assess the dose–response relationship, a series of doses spanning a wide range was generated using a concentration gradient dilution scheme. The experiment is repeated three times to enhance reliability and minimize bias.

In each trial, the plates were organized with wells containing varying drug concentrations arranged in descending order, from high to no drug (medium only), within individual rows. Each concentration was replicated three times within the same row. To calculate the dose–response curve, after 120 h, cell viability was assessed following the manufacturer’s protocol using alamarBlue (#DAL1100, Thermo Fisher Scientific). This procedure involves subtracting the treatment group’s reading from the baseline and dividing it by the control group’s reading.

#### 2.3.3. Cell Survival Assay Using Drug Combination

Cells (5000) were seeded in 80 μL of media in each well. To understand the response to a drug combination, the seeded cells underwent four different conditions: exposure to chemotherapy drugs, treatment with molecular inhibitors, a combination of chemotherapy drugs along with molecular inhibitors, and a medium-only control.

To prevent excessive motility that might overshadow the synergy effect, a low dose was chosen to specifically eliminate around 20% or fewer cells. The 20% inhibitory concentration (IC20) values were determined using measurements.

An experiment altering the order of both agents in a drug combination was conducted after an overnight culture. The first reagent was added, followed by the second reagent after 4 h. For the single-agent control, medium was added to ensure a consistent solution volume in each well.

After 120 h of incubation, cell viability was measured using alamarBlue (#DAL1100, Thermo Fisher Scientific) following the manufacturer’s protocol. As mentioned earlier, the control group without drug treatment was established. Calculation of the drug effect, including both the single drug effect and the combination group, is the same as the single drug effect parts. To identify the synergy effect in the combination group, we added the two single effects together and then compared them with the drug combination group using a *t*-test.

### 2.4. Genome-Wide CRISPR-Cas9 Screening of Chemoresistance and Data Analysis

#### 2.4.1. Construction of TKOv3 Library

We acquired the Toronto Knockout CRISPR (TKOv3) library, which contains 71,090 sgRNAs targeting 18,049 protein-coding genes, from Addgene (Watertown, MA, USA) and applied electroporation to expand the library 1000-fold. For lentivirus production, 7.5 × 10^6^ 293T cells were seeded in 15 cm plates and prepared for transfection. Following the manufacturer’s guidelines [41], packaging vectors psPAX2, pMD2.G (Addgene), TKOv3 library plasmid, and Lipofectamine (Thermo Fisher Scientific) were mixed in Opti-MEM™ (Thermo Fisher Scientific). After 48 h of incubation, the lentivirus-containing medium was collected and stored at −80 °C.

#### 2.4.2. Genome-Wide Pooled sgRNA Screens

MDA-MB-231 cells were transduced with the TKOv3 lentivirus library at a low MOI of 0.3. After 72 h of puromycin (2 μg/mL) selection, surviving cells were considered baseline samples (T0), and 3 × 10^7^ cells were harvested and stored at −80 °C. The remaining cells were divided into three groups (control, cisplatin treatment (1.5 µM), and doxorubicin treatment (0.026 µM)), each performed in triplicate. Following four weeks (equivalent to a single chemotherapy cycle) of treatment, 3 × 10^7^ cells were harvested from each group. Genomic DNA was extracted using the QIAamp DNA Blood Maxi Kit (Qiagen, Hilden, Germany). Two polymerase chain reactions (PCRs) were carried out to enrich the sgRNA-targeted genomic regions and amplify the sgRNA. The resulting libraries were sequenced on a NovaSeq 6000 system (Illumina, San Diego, CA, USA), producing nearly 80 million reads per sample to achieve 600× coverage of the CRISPR library.

#### 2.4.3. Analysis of CRISPR Screening Data

We used the MAGeCK algorithm to analyze genome-wide CRISPR/Cas9 knockout screening data [42]. Several statistics were used as quality control for CRISPR experiment sequence data, including mapping ratio (i.e., the ratio of mappable reads to the sgRNA library), missed sgRNA, Gini-index, within-time-point replicate correlation, and between-time-point correlation. sgRNA data were first normalized using a list of non-targeting control sgRNAs. Gene essentiality scores (beta-scores) were then determined between time points Tend and T0 in each condition, including control, cisplatin, and doxorubicin, using the MAGeCK maximum likelihood estimation method [43]. It provides a *p*-value for a gene’s impact on the cell viability phenotype. We also conducted principal component analysis using the stats R package and Pearson correlation analysis using the corrplot R package [44].

### 2.5. Bulk RNA Sequencing

We employed bulk RNA sequencing to analyze the transcriptome profiles of the MDA-MB-231 cells. Total RNA was extracted using the TRIzol™ reagent (Thermo Fisher Scientific, Waltham, MA, USA) according to the manufacturer’s instructions, with an additional DNase I digestion step to eliminate potential genomic DNA contamination. We used the Agilent 2100 Bioanalyzer (Agilent Technologies, Santa Clara, CA, USA) to assess the quality of RNA and the Qubit RNA Assay Kit (Thermo Fisher Scientific, Waltham, MA, USA) to evaluate concentration. Only high-quality RNA samples (RNS integrity number [RIN] ≥ 7.0) were used for library preparation. RNA sequencing libraries were prepared using the Illumina TruSeq^®^ Stranded mRNA Library Prep Kit (Illumina, San Diego, CA, USA) following the manufacturer’s protocol. The prepared libraries were quantified by qPCR, and their quality was verified on an Agilent 2100 Bioanalyzer. Sequencing was conducted on an Illumina NovaSeq 6000 platform, generating 75 bp single end reads. Raw reads were processed to remove adapters and low-quality bases using Trimmomatic and aligned to the reference genome using STAR aligner. Quantification of gene expression levels was performed using the featureCounts tool from the Subread package (Subread v2.0.2).

### 2.6. CRISPR-Cas9 Gene Combination Double-Knockout Screening

#### 2.6.1. Selection of Candidate Genes

For the construction of our CDKO experiment, we used three criteria to select candidate genes: (i) they were selected from the 149 hallmark genes identified in our previous research using the XDeathDB database [23]; (ii) they demonstrated high expression, defined as read counts above 50, in the MDA-MB-231 cell line based on bulk RNA-sequencing data; and (iii) they represented essential genes in genome-wide screening in the MDA-MB-231 cell line. As a result, we selected 65 candidate genes for the experiment.

#### 2.6.2. Library Construction

We developed the CDKO screening library following our recently published protocol [45]. To ensure screening quality, we included three sgRNAs per gene and selected three corresponding sgRNAs in our library for each of the 65 genes. In addition to the primary gene set, we included six genes known to form well-established synthetic lethal pairs as positive control gene pairs—PARP1 and BRCA1, PARP1 and BRD4, BRD4 and CHK1, and WEE1 and HDAC1. This design led to 71 genes in total. In selecting their sgRNAs, we referred to several widely used CRISPR libraries, such as the TKO v3 [46], human genome CRISPR knockout (hGECKOv2) [21], and human kinome CRISPR knockout (KinomeKO) named Brunello. We also considered the Vienna Bioactivity CRISPR (VBC) scores [47] in selecting sgRNA. As negative controls in the pooled library, we included 17 safe sgRNAs (8% of the total) that target non-functional regions of the genome.

The CDKO library, comprising 52,900 sgRNAs targeting 5041 gene–gene pairs, was developed and amplified 1000-fold using the electroporation method. For lentivirus production, five million 293T cells were seeded in 15 cm plates and prepared for transfection. The packaging vectors psPAX2 and pMD2.G (Addgene), cell-death CDKO library plasmid, and Lipofectamine (Thermo Fisher Scientific) were combined in OptiMEM (Thermo Fisher Scientific). After 48 h of incubation, the lentivirus-containing medium was harvested and stored at −80 °C.

#### 2.6.3. Pooled sgRNA Screening

MDA-MB-231 cells were infected with the cell-death CDKO lentiviral library at a low MOI of 0.3, and following 72 h of treatment with 5 μg/mL puromycin, the surviving cells were designated as baseline samples (T0), and 3 × 10^7^ cells were gathered and preserved at −80 °C. The remaining cells were split into three separate groups, and after a 28-day cultivation period, 3 × 10^7^ cells were collected from each group, Tend and T0, and genomic DNA was isolated using the QIAamp Blood Maxi Kit (Qiagen, Hilden, Germany). A series of two polymerase chain reactions (PCRs) were conducted to enrich sgRNA-targeted genomic regions and amplify the sgRNA, and the derived libraries were sequenced on a NextSeq 500 system (Illumina), generating nearly 10 million reads per sample and attaining 200× coverage of the cell-death CDKO library.

#### 2.6.4. CDKO CRISPR Sequencing Data Analysis

To analyze the sequencing data from the CDKO CRISPR screening, we first used FastQC to obtain an overview of basic quality control metrics for the raw next-generation sequencing data, including mapping ratio (i.e., the ratio of mappable reads to the sgRNA library), missed sgRNA, the Gini-index, within-time-point replicate correlation, and between-time-point correlation.

We calculated the LFC for each sgRNA pair using MAGeCK robust rank aggregation (RRA) [42]. We calculated SL scores using multiple methods, including GEMINI score [48], Horlbeck score [49], MAGeCK score [50], median score with and without background normalization (B/NB), and sgRNA-derived score-B/NB. We proposed the last two scores in our recently developed Synthetic Lethality Knowledgebase (SLKB) [51]. For each method, we identified the top 10% of gene pairs as potential synthetic lethal pairs and then focused on gene pairs that were consistently identified across different methods, selecting the most overlapping pairs for further study.

## 3. Results

### 3.1. Hierarchical Clustering Analysis of TNBC Transcriptome Profiles Between Patients and Cell Lines

**Figure 1:**
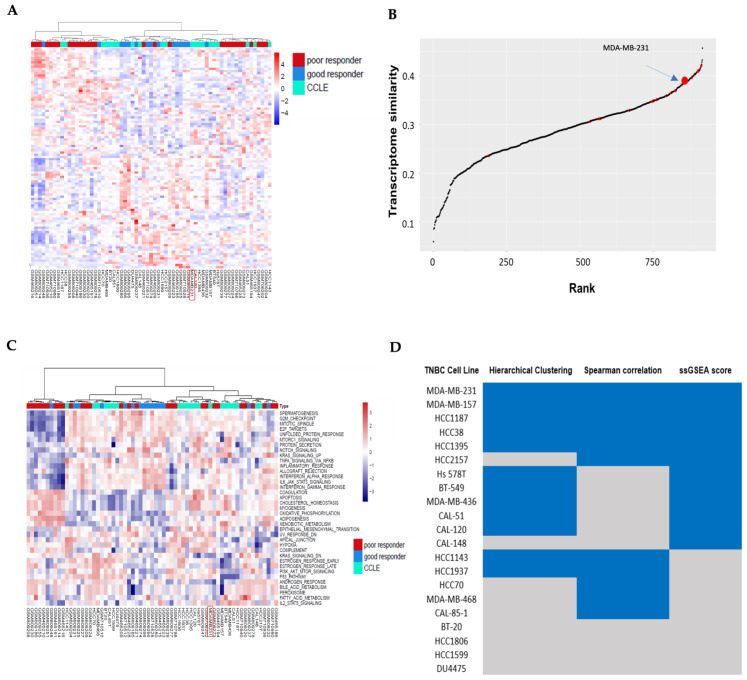
Analysis of genomic similarity between samples of patients with triple-negative breast cancer (TNBC) (baseline) and those cell lines archived in the Cancer Cell Line Encyclopedia (CCLE). (**A**) Hierarchical clustering analysis. (**B**) Spearman’s rank correlation of 916 CCLE cell lines with TNBC samples showing a poor response to chemotherapy. (**C**) Heatmap of single-sample Gene Set Enrichment Analysis (ssGSEA) scores for the 50 hallmark gene sets of the Molecular Signals Database (MSigDB) across TNBC patient samples and TNBC cell lines. (**D**) Summary table of the most representative cell line models from these three analysis methods. Similarities among cell lines in blue with those TNBC samples responding poorly to chemotherapy showed their significant representativeness for those cells.

The first clustering analysis was performed between TNBC baseline transcriptome samples and CCLE TNBC cells. In comparing chemotherapy responders to non-responders, significantly upregulated or downregulated genes represented intrinsic chemoresistance mechanisms. Statistically significantly differentially expressed genes were chosen using the fold change greater than 1.5 or less than −1.5 and adjusted *p*-value less than 0.01 (Supplementary Table S1 DEG gene list). We then used these gene expression data to perform hierarchical clustering analysis on both TNBC patient samples and TNBC cell lines. Clustering analysis in Figure 1A revealed distinct patterns between chemotherapy responders and non-responders. Notably, some TNBC cell lines were clustered together with TNBC non-responders. It suggests these cell lines as representative models for intrinsic chemo-resistance TNBC patient samples.

**Figure 2:**
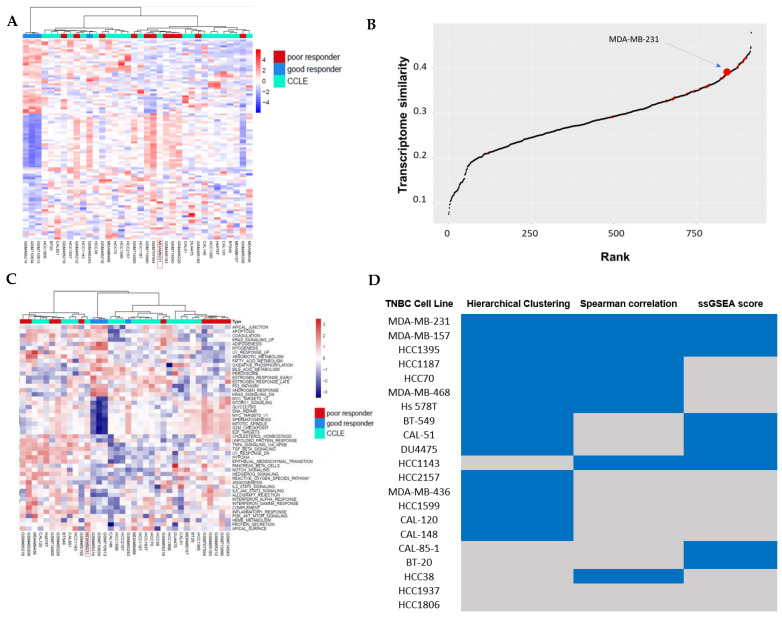
Genomic similarity analysis between samples of patients with triple-negative breast cancer (TNBC) following chemotherapy and the Cancer Cell Line Encyclopedia (CCLE) cell line model: (**A**) Hierarchical clustering analysis. (**B**) Spearman’s rank correlation of 916 CCLE cell lines with TNBC samples, showing a poor response to chemotherapy. (**C**) Heatmap of single-sample Gene Set Enrichment Analysis (ssGSEA) scores for the 50 hallmark gene sets of the Molecular Signals Database (MSigDB) across TNBC patient samples and TNBC cell lines. (**D**) Summary table of the most representative cell line models from these three analysis methods. Cell lines marked in blue indicate significant representativeness for TNBC poor responders.

The second clustering analysis was performed between TNBC transcriptome samples after chemotherapy and CCLE TNBC cells. In comparing chemotherapy responders to non-responders, significantly upregulated or downregulated genes represented acquired chemoresistance mechanisms in TNBC patients. Similarly to the first clustering analysis on the baseline TNBC transcriptome data, this cluster analysis, as shown in Figure 2A, showed clear patterns between those that did and those that did not respond to chemotherapy. Interestingly, some TNBC cell lines were again clustered with the non-responders, further supporting their value as representative models for studying chemo-resistance in TNBC.

### 3.2. Correlation Analysis Between TNBC Cell Lines and TNBC Non-Responders of Chemotherapy

Spearman correlation analysis was performed between the 916 CCLE cell lines and TNBC non-responders using the 2000 most varied genes. Cell lines were ranked based on their average correlation values. Figure 1B shows the correlation between TNBC cells and TNBC baseline samples that were chemotherapy non-responders, and Figure 2B displays correlations with post-chemotherapy non-responder TNBC samples. The TNBC cell HCC70 had the highest transcriptome similarity with TNBC non-responders at baseline, its Spearman rank correlation was 0.42, and its rank was 5 (Supplementary Table S2 baseline cell line correlation rank list). HCC1143 was the TNBC cell line showing the highest transcriptome similarity with TNBC non-responders following chemotherapy, its Spearman rank correlation was 0.42, and its rank was 17 (Supplementary Table S3 post cell line correlation rank list).

### 3.3. Pathway Similarity Analysis by Single-Sample Gene Set Enrichment Analysis (ssGSEA) Score and Overall Similarity Analysis Between TNBC Cell Lines and TNBC Chemotherapy Non-Responders

ssGSEA scores of 50 hallmark gene sets in the Molecular Signatures Database (MSigDB) were used to assess similarity between TNBC cell lines and TNBC chemotherapy non-responder samples at the pathway level. Their similarities were shown in hierarchical clustering analyses. Figure 1C shows the pathway similarity between TNBC cells and baseline TNBC non-responder samples, and Figure 2C displays the pathway similarity between TNBC cells and post-chemotherapy TNBC non-responder samples. Both cluster analyses revealed distinct clusters of TNBC cell lines and TNBC non-responder samples.

In transcriptome clustering analysis and pathway clustering analysis, a TNBC cell was labeled as 1 if it was grouped together with chemotherapy non-responder TNBC samples. In Spearman correlation analysis, a TNBC cell was denoted as 1 if it was ranked in the top 100 among 916 cancer cells. The overall similarity between TNBCs and TNBC chemotherapy non-responders was ranked by the sum of these three similarity labels. Figure 1D ranks the TNBC cells for their overall similarity with baseline TNBC non-responders, and Figure 2D shows the overall similarity with TNBC non-responders after chemotherapy. Strikingly, MDA-MB-231 and MDA-MB-157 ranked top two in both overall similarity analyses.

### 3.4. Genome-Wide CRISPR Screening on MDA-MB-231 Cell Lines

**Figure 3:**
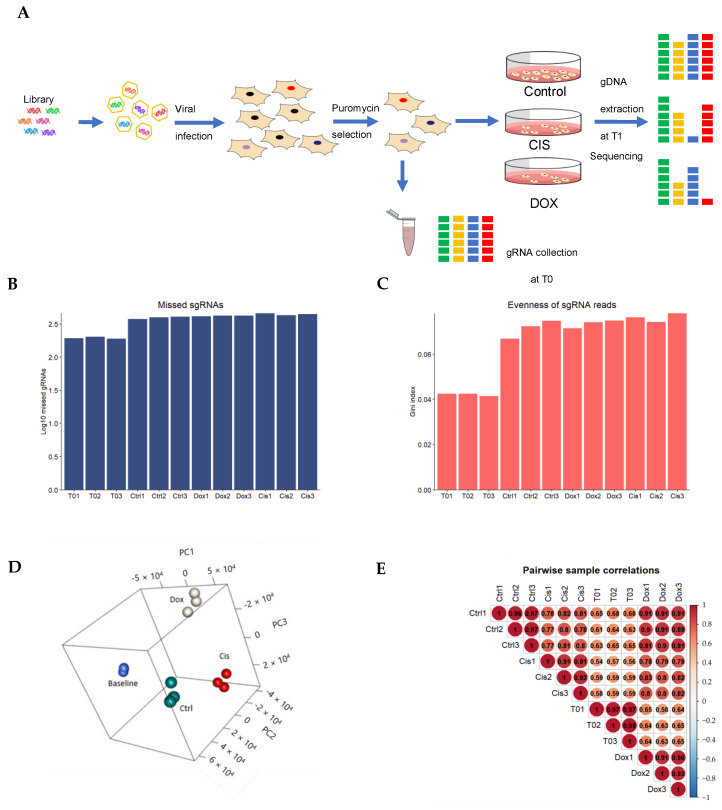
Genome-wide clustered regularly interspaced short palindromic repeats (CRISPR)-Cas9 negative screens in the MDA-MB-231 cell line. (**A**) Schematic diagram for genome-wide CRISPR by the Toronto KnockOut CRISPR library (TKOV3; Version 3) (CIS, Cisplatin; DOX, doxorubicin). (**B**) The missed sgRNAs were tested on Days 0 (T0) and 28. (**C**) Gini index of sgRNAs on Days 0 (T0) and 28. (**D**) Three-dimensional PAC plot of baseline, control (Ctrl), and two-drug treatment groups. PC1 to PC3 can explain more than 80% of the total information. (**E**) The correlation plot among baseline, control, and two-drug treatment group, each group containing triplicate samples.

A genome-wide CRISPR/cas9 screening was conducted to identify genes related to cisplatin or doxorubicin resistance in the most representative cell line, MDA-MB-231, using the TKOv3 sgRNA library, which contains 70,948 sgRNAs targeting 18,053 genes. After transducing cells with the lentiviral pooled sgRNA library with lower multiplicity of infection (MOI) (0.3) and puromycin selection, the baseline sample was harvested. On Day 28, we collected triplicate samples obtained after treatment with cisplatin or doxorubicin or treatment with DMSO (dimethyl sulfoxide) (Figure 3A). After sequencing, data analysis was performed in MAGeCK. The number of missed genes for all samples ranged between 200~400 sgRNAs among the library’s total 70,948 sgRNAs (Figure 3B), i.e., less than 0.56%. An increase in the Gini index was observed from 0.04 to 0.06 on Days 0 and 28, respectively (Figure 3C). It reflected the selective adaptation of cancer cells after CRISPR-Cas9 perturbation and consequently greater unevenness in the pooled sgRNAs due to longer treatment. From the three-dimensional principal component analysis (PCA) plot (Figure 3D) and pairwise sample correlation plot (Figure 3E), samples in the same treatment group show more similarity and higher correlation values. Overall, these quality control analyses indicated a successful genome-wide CRISPR-Cas9 screening of one MDA-MB-231 cell line.

**Figure 4:**
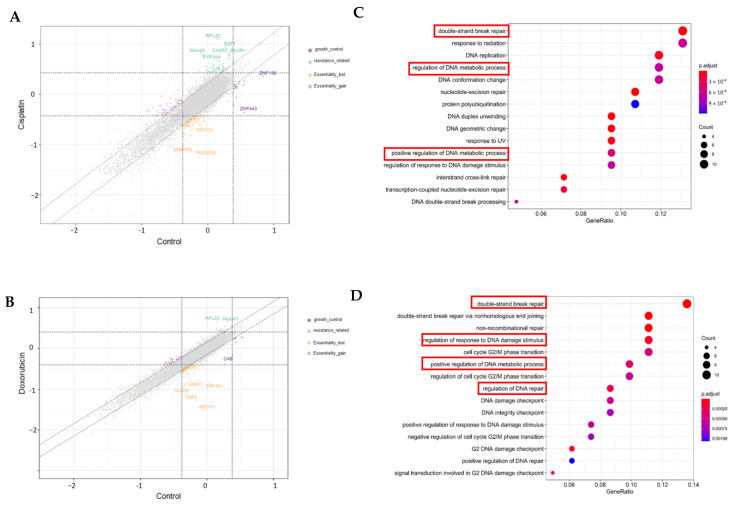
Gene essentiality scores (β scores) reported using the MAGeCK–maximum-likelihood estimation algorithm in cisplatin treatment (**A**) and doxorubicin treatment (**B**). Essential gene enrichment pathways in cisplatin treatment (**C**) and doxorubicin treatment (**D**) by analysis using the Kyoto Encyclopedia of Genes and Genomes (KEGG).

Using the MAGeCK algorithm, β scores were calculated. In the control group, a lower β score indicated a more depleted sgRNA in the final time point; in other words, gene knockout led to more cell death or was more essential to cell viability. Therefore, the β score was also called the gene essentiality score in the control group. In either cisplatin- or doxorubicin-treated groups, a lower β score indicated its corresponding gene knockout led to more cell death after chemo treatment. In other words, gene knockout re-sensitized chemo drug treatment. A lower β score within the range of −1 and +1 indicated the gene knockout did not have much influence on cell growth or cell death. In this paper, genes were selected with a β score higher than 1 in the control group but a β score less than −1 in the chemo treatment group. This means these genes were not essential in MDB-MA-231, but their CRISPR-Cas9 knockout re-sensitized low-dose chemo treatment. Figure 4A,B lay out the β scores between control and doxorubicin and cisplatin treatment, respectively. Genes in the region higher than 1 on tthhe x-axis and less than −1 on the y-axis were our selected candidate genes. We identified 96 genes that re-sensitized cisplatin treatment (Supplementary Table S4 essential gene list in cisplatin treatment) and 93 genes that re-sensitized doxorubicin treatment (Supplementary Table S5 essential gene list in doxorubicin treatment). They had 19 overlapping genes between the two lists (Supplementary Table S6 19 overlapped gene list). Kyoto Encyclopedia of Genes and Genomes (KEGG) pathway analysis was performed for two gene lists for cisplatin treatment and doxorubicin treatment, respectively. Figure 4C depicts the 15 most enriched pathways for cisplatin, and Figure 4D compares those for doxorubicin. In both chemo treatments, most enriched pathways are associated with DNA damage/DNA repair signaling pathways.

### 3.5. Validation of Targeted Gene Knockout for Increased Chemo-Sensitivity in TNBC

In this study, candidate genes were validated through the literature review. In our CRISPR-Cas9 screening, there were 96 genes that re-sensitized cisplatin treatment, and 93 genes that re-sensitized doxorubicin treatment. Among these genes, 28 were investigated and published in vivo or in vitro studies on cisplatin- or doxorubicin-resistance (Table 2). After filtering out those previously studied genes, only druggable genes were considered in our follow-up analysis. That is, the candidate gene must be a viable drug target, aligning with our objective to discover drug combinations that can overcome chemoresistance in TNBC. This analysis led to several candidate genes, including *ERCC1, NFE2L2, PRKCG, ATR, NEPPS,* and *MCM9*, for testing.

**Figure 5:**
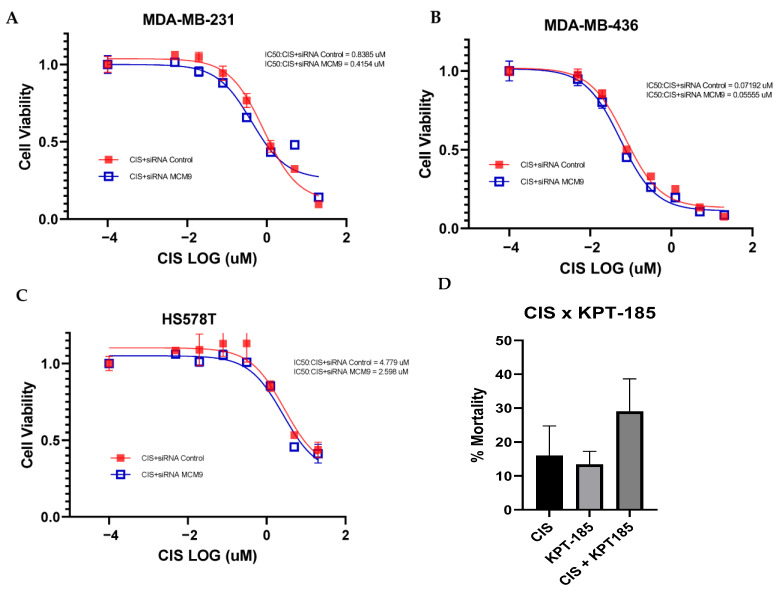
Cell growth inhibition of MDA-MB-231, MDA-MB-436, and HS578T transfected siRNA MCM9, followed by treatment with four serially diluted cisplatin doses for 120 h in (**A**–**C**). (**D**) Drug combination study between cisplatin and MCM9 inhibitor KPT-185.

We performed a cell survival assay using siRNA-mediated gene silencing to validate the novel genes identified from our CRISPR essential gene list and observed that the expression of those six genes was knocked down, but only one gene was validated in the siRNA experiment, namely *MCM9*. In Figure 5A–C, gene knockdown by siRNA led to an increase in cisplatin sensitivity of three TNBC cell lines, MDA-MB-231, MDA-MB-436, and HS578T.

Small molecule KPT-185 specifically targets the protein expressed by *MCM9*. Drug combination assays were performed using cisplatin and the *MCM9* inhibitor KPT-185. After the IC20 values for cisplatin (1.5 µM) and KPT-185 (29.6 µM) were determined through cell dose–response curve assays, combination assays of IC20 doses were evaluated. In MDA-MB-231 cell, an additive effect between the MCM9 inhibitor KPT-185 and cisplatin was observed (Figure 5D).

### 3.6. CRISPR-Cas9 Gene Combination Double-Knockout (CDKO) Experiment

**Figure 6:**
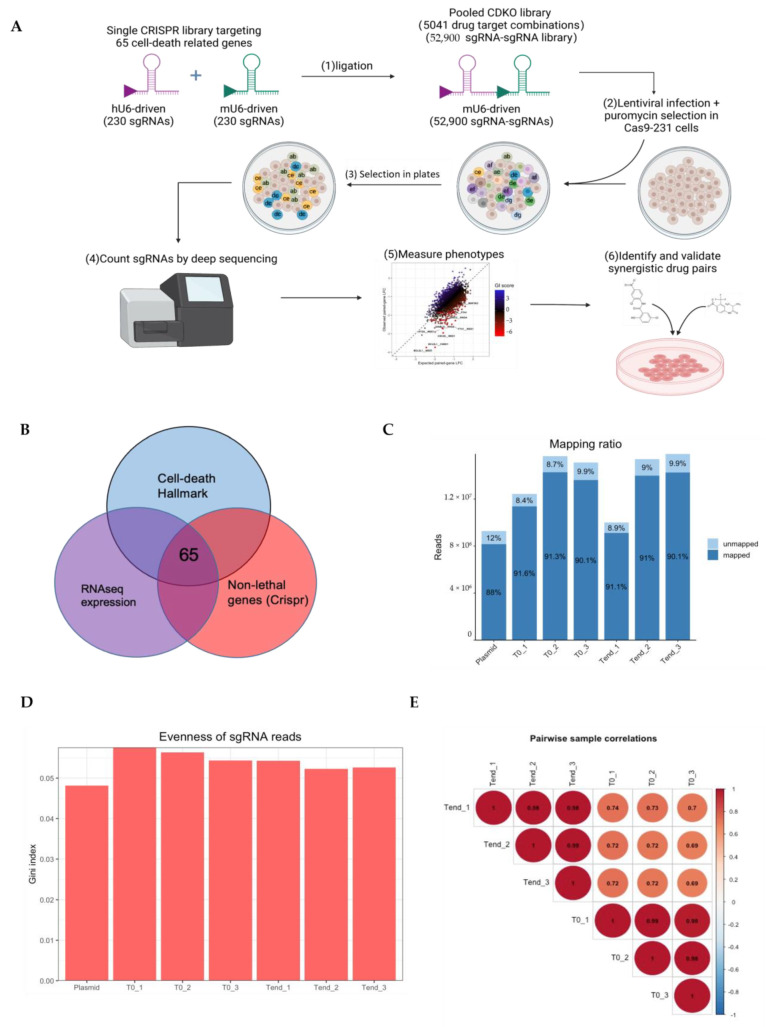
Cell-death clustered regularly interspaced short palindromic repeats (CRISPR)-Cas9 double-knockout screens in the MDA-MB-231 cell line. (**A**) Schematic diagram for genome-wide CRISPR by the cell-death CDKO library. (**B**) Candidate gene selection for constructing the cell-death CDKO library. (**C**) Read counts Mapping ratio of plasmid, days 0 (T0), and days 28 (Tend) samples. (**D**) The Gini index of sgRNAs on plasmid, days 0 (T0), and days 28 (Trend) samples. (**E**) Correlation plot between the baseline Day 0 (T0) and end date Day 28 (Tend) groups, each group containing triplicate samples.

Candidate genes were selected according to their overlap from three datasets, as shown in the Venn diagram plot in Figure 6B. These 65 genes were selected among cell death hallmark genes, expressed genes in the MDA-MB-231 cell line, and non-essential in genome-wide CRISPR-Cas9 screening. Table 3 details these 65 selected genes, including their functions and relevant cell-death modes.

A CDKO experiment was conducted to identify SL gene pairs among 65 selected genes in the MDA-MB-231 cell line (Figure 6A). Cells were transduced with the lentiviral pooled sgRNA library at a low MOI (0.3) and applied puromycin selection before collecting the baseline sample. After 28 days of cell culture, cells were harvested in triplicate samples. The sequencing results demonstrated high quality, with a mapping ratio of approximately 90% for all samples and around 10 million mapped reads per sample (Figure 6C). The number of missed genes ranged from 39 to 1000 dual-sgRNA out of a total of 52,900 sgRNAs (Table 4). The Gini index for all samples was around 0.05 (Table 4, Figure 6D). The pairwise sample correlation (Figure 6E) plots show a distinct pattern and higher correlation values of samples within the same treatment group across triplicate samples.

**Figure 7:**
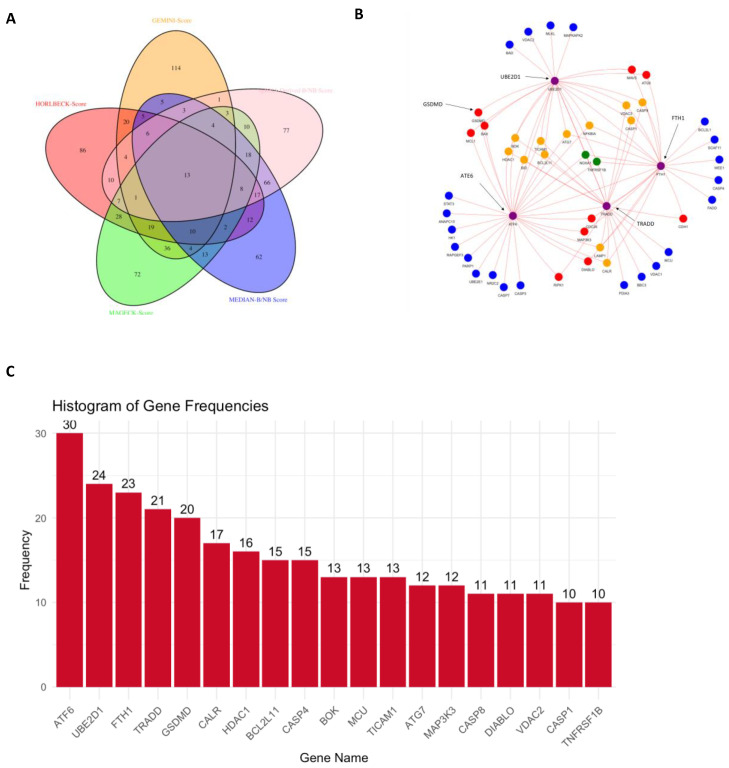
Identification of synergistic lethal (SL) gene pairs. (**A**) Venn plot of seven methods for scoring SL pairs. (**B**) Gene network by 242 SL plots. (**C**) Histogram displaying the frequency distribution of top hub genes in 242 SL gene pairs.

Five SL score calculation methods were implemented to select SL gene pairs (see Methods). Their overlaps are depicted in both the Venn diagram (Figure 7A). Among 2080 screened gene pairs, 242 gene pairs exhibited SL in more than three methods and are more likely than others to have an SL effect. An SL network based on these 242 gene pairs is shown in Figure 7B. Hub genes interacting with at least ten other genes are displayed in the histogram (Figure 7C).

## 4. Discussion

### 4.1. CRISPR-Cas9 Gene Combination Double-Knockout (CDKO) Experiment

For the first time, representative chemo-resistance TNBC cell line models were selected. It was based on three clustering/correlation analyses in the transcriptome between TNBC cell lines and TNBC chemotherapy patient responders and non-responders. The first analysis was based on a set of differentially expressed genes between chemotherapy responders and non-responders. The cluster analysis reveals TNBC cell lines that were grouped with non-responders. The second analysis started from 2000 highly varied genes among 916 cancer cell lines. Using these 2000 genes, it ranked 916 cancer cell lines in Spearman correlations with TNBC chemotherapy non-responders. The top TNBC cells were selected based on this ranking. Although these two analyses both used correlations between cell lines and TNBC non-responder patient samples, they differed in how genes were selected for correlation analysis. The first analysis selected genes from patient samples, while the second selected genes from cell lines. They are complementary to each other. The third analysis, on the other hand, focused on correlation analysis based on pathway enrichment on cancer hallmark gene sets in the Molecular Signatures Database (MSigDB) [37]. These gene sets are directly related to cancer development and progression, including cell cycle regulation, apoptosis, DNA repair, epithelial–mesenchymal transition (EMT), hypoxia, and others. Furthermore, when studying chemotherapy non-responders, transcriptome data at baseline (i.e., intrinsic chemo-resistance) and transcriptome data after chemotherapy (i.e., acquired chemo-resistance) were both considered in all three TNBC cell correlation/cluster analyses. In our view, these analyses provided the most comprehensive view of the similarities between TNBC cells and TNBC chemotherapy non-responders. We found the MDA-MB-231, MDA-MB-157, HCC1187, HCC38, and HCC1395 cell lines to be the most representative across all three methods. Three of these, MDA-MB-231, MDA-MB-157, and HCC1395, were consistently significant and therefore most representative across the three methods when we analyzed similarity with samples of TNBC poor responder samples following chemotherapy.

### 4.2. TNBC Chemo-Resistant Target Discovery Through Genome-Wide CRISPR-Cas9 Screening and Validation Analyses

We used genome-wide CRISPR screening to identify essential genes that could help overcome resistance to cisplatin and doxorubicin (Table 2) in triple-negative breast cancer. *DNMT1, PPIA, RUNX, BCL2L1, RUNX2, NBN, GTF2H5, USP22, HSP90AB1, CDC25B, NCF1, FANCA, FANCG,* and *ERCC1* are well-studied genes for cisplatin treatment, some having known drug targets or inhibitors. *DNMT1*, for example, is involved in the DNA methylation pathway and targeted by decitabine, which is used clinically to treat other types of cancer [138]. In addition, *RUNX*, which regulates apoptosis and cell proliferation, has a small molecule inhibitor (AI-10-49) in preclinical development; *BCL2L1*, involved in the intrinsic apoptotic pathway, can be targeted by BikDD and lapatinib, both in preclinical investigation [138]; and *RUNX2*, which also plays a role in regulating the cell cycle and apoptosis, is targeted by BET inhibitors JQ1 and I-BET762, which are currently in Phase I/II clinical trials [139].

Other genes in our list have been implicated in chemoresistance in breast and other cancer cells. *PPIA* participates in the regulation of miRNA and impacts the sensitivity of breast cancer cells to doxorubicin [140] and the knockdown of *RUNX*, which is involved in the YAP signaling pathway, enhances sensitivity to doxorubicin in breast cancer cells [139]. In HER2- and MDM2-enriched breast cancer subtypes, *NBN* plays a role in doxorubicin, paclitaxel, and carboplatin resistance via its involvement in DNA repair and homologous recombination. Involved in nucleotide excision repair (NER), *GTF2H5* has been shown to predict survival in high-grade serous ovarian cancer.

For doxorubicin treatment, our gene list includes *ABCC1, HIST1H2BJ, ZEB2, ATM, FANCL, CDC25B, XRCC1, ACTG1, IRS1, NBN, NFE2L2, NDUFB9, CDK5,* and *CDCA3*, some of which are reported to play a role in chemoresistance. In TNBC, for instance, *ABCC1* is a drug efflux transporter implicated in resistance to doxorubicin, paclitaxel, and cisplatin [141], and the involvement of *HIST1H2BJ* in glutathione synthesis and copper chelation promotes resistance to doxorubicin, paclitaxel, and cisplatin [142]. *ZEB2*, a transcription factor, is associated with drug resistance in breast cancer cells through its regulation of the epithelial–mesenchymal transition (EMT) [143].

Some of these genes have potential drug targets or inhibitors. The ATM kinase inhibitor KU-55933, which targets ATMs involved in the DNA damage response pathway, is in preclinical development [144]; *FANCL*, part of the Fanconi anemia DNA repair pathway, has been targeted by small molecule inhibitors, such as curcumin, in preclinical studies [145]; and *CDK5* inhibitors, like roscovitine and dinaciclib, have shown promise in preclinical studies and are in clinical trials for various cancer types [146]. *CDK5* is involved in cell cycle regulation and the DNA damage response, which contribute to chemoresistance.

*CDC25B* and *NBN* are the overlapping genes between the cisplatin and doxorubicin essential gene lists. *CDC25B* is involved in cell cycle regulation and DNA damage response and has been targeted by thiostrepton, FDI-6, and siomycin A in preclinical studies for the treatment of platinum-resistant ovarian cancer [142]. *NBN*, on the other hand, is involved in DNA repair and homologous recombination and has been studied in vitro using siRNA. Though no drug targets or inhibitors have been identified for NBN, its role in DNA repair suggests it may be a potential therapeutic target in the future.

In validating some of the new targets that are druggable, we employed the MDA-MB-231 cell line used for our initial screening, as well as such other TNBC cell lines such as MDA-MB-436 and HS578T. Our results showed a significant reduction in cell viability in all three cell lines following the knockdown of *MCM9*, indicating an increased sensitivity to cisplatin treatment. Our study also identified an additive effect of KPT-185, an *MCM9* inhibitor, when combined with cisplatin in in vitro assays. Cisplatin is a commonly used chemotherapeutic drug in TNBC. Its mode of action when entering cancer cells is to bind to DNA, causing DNA crosslinks and DNA damage, subsequently inducing apoptosis in cancer cells [4,147]. *MCM9* can combine with *MCM8* to form a complex, which is required for DNA damage repair caused by DNA interstrand crosslinks [148,149]. Therefore, *MCM9* inhibition could sensitize TNBC cells to Cisplatin. The intersection of these methods aids the confident identification of the core set of essential genes, such as *MCM9*, as potential gene targets to overcome triple-negative breast cancer.

Doxorubicin and cisplatin exert cytotoxic effects through distinct mechanisms; therefore, the relatively low overlap in re-sensitizing genes is expected. In this study, our primary aim was to identify the common factors involved in overcoming chemotherapy resistance in triple-negative breast cancer. In future work, we plan to investigate drug-specific resistance mechanisms.

### 4.3. Discovery of Synthetic Lethal Gene Pairs in TNBC Cells

We introduced five SL gene pair as positive controls, four of which were ranked within the top 10% based on their LFC ranking scores according to the results from our screening data analysis (*BRD4_PARP1*, rank 9, SL score 8.56 × 10^−10^; *HDAC1__WEE1*, rank 124, SL score 3.29 × 10^−5^; *BRD4__CHEK1*, rank 172, SL score 0.00016564; and *HDAC2__WEE1*, rank 200, SL score 0.00027904). The presence of these positive control gene pairs in our results demonstrates the success of our experiment. *HDAC1* inhibition has been shown to induce histone tail acetylation and an open chromatin structure, which promotes the expression of cell differentiation or death genes. In combination with *WEE1* inhibition, impaired activity of the cell cycle checkpoint kinase and premature mitotic entry lead to an increase in DNA damage and apoptosis, making this combination a promising target for the treatment of acute myeloid leukemia (AML) [150]. Similarly, inhibition of *BRD4* has been shown to induce homologous recombination deficiency and sensitize cells to inhibitors of poly(ADP-ribose) polymerases (PARPis). This combination has the potential to reverse intrinsic resistance in RAS-mutant tumors and other mechanisms of PARPi resistance, warranting clinical assessment in both PARPi-sensitive and -resistant cancers [151].

Our findings add to the ongoing efforts to discover novel synthetic lethal interactions that can be harnessed for targeted cancer therapy. The 242 synthetic lethal pairs identified in this study offer a valuable resource for future research in TNBC and other cancer types. Some of the 242 gene pairs we identified have already been reported to demonstrate synthetic lethal effects. The *CDH1–HDAC1* and *HDAC1–PARP1* gene pairs have been reported as synergistically lethal gene pairs and can be considered potential drug targets. Decourtye-Espiard and colleagues investigated the potential of histone deacetylase (HDAC) inhibitors as a treatment for cancers with *CDH1* mutations, testing the effects of several HDAC inhibitors on gastric and breast preclinical models with and without *CDH1* mutations [152], and observed greater sensitivity of *CDH1*-null cells than wild-type cells to pan-HDAC inhibitors, such as entinostat, pracinostat, mocetinostat, and vorinostat. This supports the notion that *CDH1-HDAC1* has a strong synergistic lethal effect on cancer. In our study, we identified another potential synthetic lethal gene pair, *HDAC1* and *VDAC2*. *VDAC2* has been reported to be closely related to apoptosis. When VDAC2 is inhibited, VDAC2 releases BAK, allowing it to oligomerize and trigger mitochondrial outer membrane permeabilization (MOMP), which leads to apoptosis [153,154]. Therefore, dual loss of *VDAC2* and *HDAC1* is likely to enhance apoptosis, providing a plausible basis for synthetic lethality. The combination of hypomethylating agents (HMAs), PARPis, and histone deacetylases (HDACis) was hypothesized to be synergistically cytotoxic to leukemia and lymphoma cells. Valdez’s research team demonstrated that exposing AML and lymphoma cell lines to a combination of PARPi niraparib, HMA decitabine, and HDACi romidepsin or panobinostat led to a synergistic inhibition of cell proliferation up to 70%. This combination activated the ATM pathway, increased the production of reactive oxygen species, decreased mitochondrial membrane potential, and induced apoptosis [152]. This provides robust evidence that our CRISPR double-knockout experiments are indeed effective in identifying synthetic lethal gene pairs.

In the gene–gene network focusing on 242 SL interactions, we specifically selected hub genes of interest, those with the highest number of pairs (Figure 7C). Six of the nineteen hub genes were found to have related drug inhibitors, most identified in previous research for TNBC treatment (Table 5). However, though individual drugs have been studied, no research on drug combinations is reported. This provides us with ample opportunity to discover the most novel and effective drug combinations for treating triple-negative breast cancer (TNBC).

Although our study successfully identified synthetic lethal gene pairs in the MDA-MB-231 cell line, further validation of these interactions in additional TNBC cell lines and in vivo models is necessary to confirm their relevance and potential as therapeutic targets. This requires more validation experiments, such as designing a CRISPR screening library that targets only the 242 known gene pairs and assaying two-drug combinations. To understand the underlying molecular mechanisms driving synthetic lethality and determine the optimal drug combinaftions and dosages for clinical translation, detailed functional characterization of the identified gene pairs is also needed.

## 5. Conclusions

In conclusion, our CRISPR-based screening approach has successfully identified synthetic lethal gene pairs in a representative TNBC cell line, offering new potential drug targets for the treatment of this aggressive and challenging cancer subtype. Future studies should focus on validating these interactions in additional models and characterizing their functional roles to pave the way for the development of innovative targeted therapies for patients with TNBC.

Our study strongly supports the notion that utilizing CRISPR screening for essential cancer genes is an efficient and practical approach to identify potential drug targets to overcome resistance to chemotherapeutic drugs. By integrating multiple data analysis techniques and biological experimental analyses, we have identified several genes that hold promise as potential drug targets for combating chemoresistance. Our findings underscore the importance of leveraging advanced genetic screening tools and data-driven methods to better understand the molecular mechanisms underlying drug resistance and to develop more effective therapeutic strategies for cancer treatment.

## Figures and Tables

**Table 1:** Genomic dataset in study of chemoresistance in breast cancer and genomic profiles of Cancer Cell Line Encyclopedia (CCLE) cell lines.

Dataset	Sample Size	Platform	Source	Publication	Genes and Pathways Discovered in Breast Cancer Chemoresistance
GSE28844	61	Affymetrix (Affymetrix, Santa Clara, CA, USA)	Patient	Laura et al., 2013 [31]	chemoresistance-associated gene shows enrichment in Wnt, HIF1, p53, and Rho GTPases signaling pathways
GSE18728	61	Affymetrix (Affymetrix, Santa Clara, CA, USA)	Patient	Korde et al., 2010 [32]	MAP-2, MACF1, VEGF-B, EGFR showed upregulation in poor responder after chemotherapy
GSE32646	115	Affymetrix (Affymetrix, Santa Clara, CA, USA)	Patient	Miyake et al., 2012 [33]	GSTP1 expression predicts poor response to neoadjuvant chemotherapy in patient with ER-negative breast cancer
GSE36133 (CCLE)	917 (21 TNBC)	Affymetrix (Affymetrix, Santa Clara, CA, USA)	Cell line	Barretina et al., 2010 [30]	.

**Table 2:** Well-studied genes in the essential gene list for doxorubicin and cisplatin treatment. BET, bromodomain and extra-terminal motif proteins BRD2, BRD3, BRD4, and BRDT; ROS, reactive oxygen species.

Gene	Pathway	Validation Method	Inhibitor	Drug Development Status	Chemotherapy Drug	Cancer Type
CDC25B [52]	Cell cycle regulation, DNA damage response	In vitro (siRNA), In vivo (xenograft model)	Thiostrepton, FDI-6, Siomycin A	Preclinical	Paclitaxel, Cisplatin	Platinum-resistant ovarian cancer
NCF1 [53]	Autophagy, ROS production	In vitro (siRNA)	Ginsenoside Ro	Preclinical	5-fluorouracil	Esophageal cancer
USP22, HSP90AB1 [54]	HSP90 regulation and ubiquitin pathway	In vitro (siRNA), In vivo (xenograft model)	Ganetespib, AT13387	Phase II trials	Irinotecan	Mammary and colorectal cancer
DNMT1 [55]	DNA methylation	In vitro (siRNA), In vivo (mice model, xenograft with gene knockdown)	Decitabine	Clinical (used for other cancer types)	Decitabine	Triple-negative breast cancer
BCL2L1 [56]	Apoptosis	In vitro (cell lines), In vivo (mice model)	BikDD, Lapatinib	Preclinical	Doxorubicin	breast cancer
RUNX2 [57]	BET inhibition	In vitro (siRNA), In vivo (xenograft model, CRISPR knockout)	BET inhibitors: JQ1, I-BET762	Preclinical, Phase I/II	Cisplatin, Taxanes	Triple-negative breast cancer
HSP90 [58]	Chaperone protein function	In vitro (siRNA), In vivo (xenograft model)	17-AAG, PU-H71	Phase II/III trials	Doxorubicin	HER2-negative breast cancer
PPIA [59]	miRNA regulation	In vitro (miRNA-192-5p mimic)	-	-	Doxorubicin	Breast cancer
RUNX1 [60]	YAP signaling pathway	In vitro (shRNA knockdown), In vivo (xenograft)	-	-	Doxorubicin	Breast cancer
NBN [61]	DNA repair, homologous recombination	In vitro (siRNA)	-	-	Doxorubicin, Paclitaxel, Carboplatin	HER2- and MDM2-enriched breast cancer subtypes
GTF2H5 [62]	Nucleotide excision repair (NER)	In vitro	-	-	Carboplatin, Paclitaxel	High-grade serous ovarian cancer
FANCA, FANCG [63]	DNA damage repair, Fanconi anemia/BRCA pathway	In vitro (siRNA)	-	-	Cisplatin	Drug-resistant lung cancer
ERCC1 [64]	Nucleotide excision repair	In vitro (siRNA), In vivo (xenograft model)	-	-	Cisplatin	Various cancer types
XRCC1 [65]	DNA repair	In vitro (siRNA)	Triptolide	Preclinical	Cisplatin	Triple-negative breast cancer
XRCC1 [66]	Base excision repair	In vitro (siRNA)	Berberine	Preclinical	Epirubicin, Doxorubicin, Cyclophosphamide, 5-fluorouracil, Docetaxel, Cisplatin	Breast cancer
IRS1 [67]	PI3K-AKT-mTOR signaling	In vitro (miRNA and inhibitor)	Y-29794	Preclinical	Paclitaxel, Carboplatin, Gemcitabine, Doxorubicin, Cisplatin	Triple-negative breast cancer
Cdk5 [68]	Cell cycle regulation, carboplatin-induced cell death	In vitro (siRNA)	-	-	Carboplatin	Breast cancer
FANCL [63]	Fanconi anemia/BRCA pathway	In vitro (siRNA)	-	-	Cisplatin	Lung cancer
NFE2L2 [69]	Chemotherapy resistance, hypoxia response	In vitro (siRNA, hypoxia exposure)	-	-	Cisplatin, Doxorubicin, and Etoposide	Breast cancer
NBN [61]	Homologous recombination DNA repair	In vitro (immuno-fluorescence, Western blot)	-	-	Docetaxel, Doxorubicin, and Cyclophosphamide	Breast cancer
HIST1H2BJ [70,71]	Glutathione synthesis, copper chelation	In vitro (siRNA), In vivo (mice)	-	-	Doxorubicin, Paclitaxel, 5-fluorouracil	Breast cancer
ABCC1 [72]	Drug efflux transporters	In vitro (siRNA)	-	-	Doxorubicin, Paclitaxel, Cisplatin	Triple-negative breast cancer
ZEB2 [73]	ATM activation	In vitro (siRNA)	-	-	Doxorubicin, Paclitaxel, Cisplatin	Breast cancer
CDK5 [74]	Drug resistance-related pathways	In vitro (siRNA)	-	-	Paclitaxel, Cisplatin, and Doxorubicin	Triple-negative breast cancer
CDCA3 [75]	Cell proliferation, metastasis, chemoresistance	In vitro (siRNA, RT-qPCR)	-	-	Paclitaxel, Cisplatin, and Doxorubicin	Triple-negative breast cancer
CDC25B [52]	Cell cycle regulation	In vitro (siRNA)	-	-	Paclitaxel, Cisplatin	Platinum-resistant ovarian cancer
ATM [76]	Cell cycle regulation	In vitro (siRNA), In vivo (xenograft mice)	-	-	Taxanes	Breast cancer

**Table 3:** Candidate genes for developing cell-death CRISPR double-knockout (CDKO) library.

Genes	Cell_Death_Mode	Synonyms	Reference
VDAC3	Ferroptosis	VDAC-3, HD-VDAC3, HVDAC	Lemasters 2017 [77]
VDAC2	Ferroptosis	VDAC-2, HVDAC2, POR	Lemasters 2017 [77]
ATG7	Autophagy	Ubiquitin-activating enzyme E1-like protein, ubiquitin-like modifier-activating enzyme ATG7	Gomez-Puerto et al., 2016 [78]
UBE2E1	Mitotic_CD	UBCH6	Galluzzi et al., 2018 [79]
TP53	MPT	Tumor protein 53, P53	Sung et al., 2018 [80]
MCL1	Apoptosis	TM, EAT, MCL1L1	Inuzuka et al., 2011 [81]
NR2C2	Apoptosis	TAK1, TR4	Fan et al., 2018 [82]
DIABLO	Apoptosis	SMAC, DFNA64	Chai et al., 2000 [83]
STAT3	Parthanatos	Signal transducer and activator of transcription 3 (acute-phase response factor), DNA-binding protein APRF	Li et al., 2018 [84]
BBC3	Apoptosis	PUMA, JFY1	Han et al., 2001 [85]
VDAC1	MPT	PORIN, VDAC-1	Zamarin et al., 2005 [86]
PARP1	Parthanatos	Poly [ADP-Ribose] Polymerase 1, Poly [ADP-Ribose] Synthase 1, EC 2.4.2.30, ADPRT 1, PARP-1	Jiang et al., 2018 [87]
EIF2AK3	Parthanatos	PERK, PEK, HsPEK	Cubillos-Ruiz et al., 2017 [88]
NOXA1	Apoptosis	p51NOX, NY-CO-31	Kang et al., 2012 [89]
MAPKAPK2	Apoptosis	MK2, MK-2, MAPKAP-K2	Henriques et al., 2018 [90]
MAP3K3	Efferocytosis	MAPKKK3, MEKK3	Fan et al., 2014 [91]
ERN1	Parthanatos	Inositol-requiring protein 1, Inositol-requiring enzyme 1	Rufo et al., 2017 [92]
CASP1	Pyroptosis	Inflammasome (Nalp3, Asc, Casp1)	Man et al., 2017 [93]
RIPK1	Apoptosis	IMD57, RIP, RIP1	Newton 2015 [94]
TICAM1	Apoptosis	IIAE6, TRIF, MyD88-3	Galluzzi et al., 2018 [79]
IL18	Pyroptosis	IFIF, IL-18, IL1F4	Berghe et al., 2014 [95]
CASP4	Pyroptosis	ICEREL-II, ICH-2	Casson et al., 2015 [96]
TRADD	Apoptosis	Hs. 89862	Zheng et al., 2006 [97]
MLKL	Necroptosis	hMLKL	Lawlor et al. [98]
HK1	Parthanatos	HK1-Tb, HK1-Tc, HMSNR, HXK1	Guzmán 2019 [99]
GBA	Autophagy	GLCM_HUMAN, GLUC	García-Sanz et al., 2018 [100]
FADD	Apoptosis	GIG3, MORT	Chinnaiyan et al., 1995 [101]
RAPGEF3	MPT	EPAC1, HSU79275, CAP-GEFI	Galluzzi et al., 2018 [79]
PDIA3	Parthanatos	Endoplasmic reticulum resident protein 60, protein disulfide isomerase-associated 3	Liur et al., 2019 [102]
NFKBIA	Efferocytosis	EDAID2, IKBA, MAD-3	Bredel et al., 2010 [103]
UBE2D1	Mitotic_CD	E1 (17)KB1, SFT, UBC4/5	Fujikawa et al., 2020 [104]
CDH1	Parthanatos	Epithelial cadherin, CAM 120/80, CDHE, UVO	Tang et al., 2019 [105]
GSDMD	Pyroptosis	DF5L, DFNA5L	Shi et al., 2015 [106]
PPIF	Apoptosis	CYP3, CypD, CyP-M	Baines et al., 2005 [107]
CASP3	Apoptosis	CPP32, CPP32B	Ponder et al., 2019 [108]
TNFRSF1B	Efferocytosis	CD120b, TBPII, TNF-R-II, TNFR2	Pimentel-Muiños et al., 1999 [109]
TNFRSF1A	Apoptosis	CD120a, FPF, TBP1, TNF-R, R55	Galluzzi et al., 2018 [79]
LAMP1	Parthanatos	CD107 antigen-like family member A, LAMP-1, lysosomal-associated membrane protein 1	Fennelly et al., 2017 [110]
CTSL	Lysosomal_CD	CATL1, MEP, CTSL	Sargeant et al., 2014 [111]
IL1B	Pyroptosis	Catabolin, IL-1 beta, IL1F2, pro-interleukin-1-beta	Monteleone et al., 2018 [112]
CASP6	Apoptosis	CASP3/6/7, caspase 3, 6, 7, caspase-3, -6, and -7	Gröschel et al., 2018 [113]
SCAF11	Pyroptosis	CASP11, SFRS2IP, SIP1, SRRP129	Li et al., 2022 [114]
CASP7	Apoptosis	CASP-7, CMH-1, ICE-LAP3	Rager 2015 [115]
MAVS	Pyroptosis	CARDIF, IPS-1, IPS1, VISA	Kuriakose et al., 2016 [116]
ATF6	Parthanatos	CAMP-dependent transcription factor ATF-6 alpha, activating transcription factor 6 alpha, ATF6-Alph	Serrano-del Valle et al., 2019 [117]
CALR	Parthanatos	Calregulin, CRP55, ERp60, HACBP, Grp60	Gullai et al., 2018 [118]
MCU	MPT	C10orf42, CCDC109A, HsMCU	König et al., 2016 [119]
XIAP	Apoptosis	BIRC4, API3, IPA-3, XLP2, hIAP-3	Mufti et al., 2007 [120]
BCL2L11	Apoptosis	BIM, BAM, BOD,	Alvarez et al., 2018 [121,122]
BID	Apoptosis	BID isoform L(2), BID isoform Si6, FP497	Derakhshan et al., 2017 [123]
BOK	Apoptosis	BCL2L9	D’Orsi et al., 2017 [124]
BCL2	Apoptosis	BCL2, apoptosis regulator, B-cell CLL/Lymphoma, PPP1R50, Bcl-2	Campbell et al., 2018 [125]
BCL2L1	Apoptosis	BCL-XL, BCLX, BCL2L	Chen et al., 2015 [126]
BAD	Apoptosis	BBC2, BCL2L8	Letai et al., 2002 [127]
ATG3	Autophagy	Autophagy-related protein 3, APG3L, HApg, APG3	Frudd et al., 2018 [128]
ATG5	Autophagy	Autophagy protein 5, ATG5 autophagy-related 5 homolog	Ye et al., 2018 [129]
RHOA	Parthanatos	ARH12, RHO12, ARHA	Durgan et al., 2018 [130]
BAX	Apoptosis	Apoptosis regulator BAX, Bcl-2-like protein 4, Bcl2-L-4	Ke et al., 2018 [131]
CASP8	Apoptosis	APLS2B, CAP4, FLICE	Newton et al., 2019 [132]
BIRC2	Autophagy	API1, MIHB, baculoviral IAP repeat-containing 2	Campbell et al., 2018 [133]
ANAPC7	Mitotic_CD	APC7	Shi et al., 2022 [134]
ANAPC10	Mitotic_CD	APC10, DOC1	Jin et al., 2008 [135]
CDC26	Mitotic_CD	ANAPC12, APC12	Endo et al., 2010 [136]
FAS	Apoptosis	ALPS1A, APT1, CD95, APO-1	Waring et al., 1999 [137]

**Table 4:** CRISPR double-knockout (CDKO) sequencing statistics.

Sample	Reads	Mapped	Mapped %	Zero_ Counts	Zero_ Counts%	Gini_ Index	Above_ Threshold	Above_ Threshold%
Library/Plasmid	9,272,549	8,159,448	88.00	141	0.27	0.05	48,921	92.48
T0_1	12,431,419	11,383,720	91.57	892	1.69	0.06	47,101	89.04
T0_2	15,647,051	14,281,485	91.27	722	1.36	0.06	47,669	90.11
T0_3	15,104,601	13,610,181	90.11	1371	2.59	0.05	47,547	89.88
Tend_1	10,004,498	9,114,027	91.10	109	0.21	0.05	50,658	95.76
Tend_2	15,386,447	13,994,094	90.95	48	0.09	0.05	51,875	98.06
Tend_3	15,837,762	14,264,603	90.07	39	0.07	0.05	51,866	98.05

**Table 5:** Hub genes with targeting inhibitor.

Name	Pathway	Drug Bank ID	Inhibitor Name	Previous Study in TNBC	Inhibitors of Gene Pairs
FTH1	Autophagy	DB00852	Pseudo-ephedrine	Prognostic marker [155]	YF438 (HDAC1)
HDAC1	Cell cycle	DB02546	YF438	anti-TNBC activity [156]	Pseudoephedrine (FTH1), Fostamatinib (MAP3K3), Aluminum monostearate (VDAC2), Bryostatin 1 (DIABLO)
MAP3K3	Effero-cytosis	DB12010	Fostamatinib	--	YF438 (HDAC1), Aluminum monostearate (VDAC2)
DIABLO	Apoptosis	DB11752	Bryostatin 1	anti-TNBC activity [157]	YF438 (HDAC1), Aluminum monostearate (VDAC2)
VDAC2	Ferroptosis	DB01375	Aluminum monostearate	--	Bryostatin 1 (DIABLO), YF438 (HDAC1), Fostamatinib (MAP3K3)
CASP1	Pyroptosis	DB00945	Acetylsalicylic acid	anti-TNBC activity [158]	YF438 (HDAC1), Aluminum monostearate (VDAC2), Fostamatinib (MAP3K3)

## Data Availability

https://github.com/Lilab-OSU/Data-for-TKOV3-and-CDKO.git (accessed on 26 November 2025).

## Supplementary Materials

The following supporting information can be downloaded at: https://www.mdpi.com/article/10.3390/cancers17233876/s1, Table S1: DEG gene list of Poor responder vs Good responder (Baseline); Table S2: TNBC cell line correlation score with poor responder (Baseline); Table S3: TNBC cell line correlation score with poor responder (Post Chemo); Table S4: Essential gene list in cisplatin treatment; Table S5: Essential gene list in doxorubicin treatment; Table S6: 19 overlapped gene list.

## Abbreviations

The following abbreviations are used in this manuscript:

ADCs	Antibodydrug conjugates
AML	Acute myeloid leukemia
B/NB	Score with and without background normalization
CDKO	Combination double-knockout
EMT	Epithelialmesenchymal transition
ER	Estrogen receptor
GEO	Gene Expression Omnibus
GO	Gene Ontology
GSVA	Gene set variation analysis
hGECKOv2	Human genome CRISPR knockout version 2
HDAC	Histone deacetylase
HER2	Human epidermal growth factor receptor 2
HMAs	Hypomethylating agents
KEGG	Kyoto Encyclopedia of Genes and Genomes
KinomeKO	Human kinome CRISPR knockout
MOI	Multiplicity of infection
MP	MillerPayne
MSigDB	Molecular Signatures Database
mTOR	Mammalian target of rapamycin
NACT	Neoadjuvant chemotherapy
NER	Nucleotide excision repair
PCA	Principal component analysis
PARP	Poly(ADPribose) polymerase
PARPis	Poly(ADPribose) polymerase inhibitors
PCRs	Polymerase chain reactions
PI3K	Phosphoinositide 3kinase
PR	Progesterone receptor
RMA	Robust multiarray average
RRA	Robust rank aggregation
SL	Synthetic lethality
SLKB	Synthetic Lethality Knowledgebase
ssGSEA	Singlesample Gene Set Enrichment Analysis
STR	Short tandem repeat
TKOv3	Toronto Knockout CRISPR library version 3
VBC	Vienna Bioactivity CRISPR
