# Supplementary material for: CRISPR-Cas9 Genome and Double-Knockout Screening to Identify Novel Therapeutic Targets for Chemoresistance in Triple-Negative Breast Cancer
Source: Cancers (Basel). 2025 Dec 3;17(23):3876. doi: 10.3390/cancers17233876 (PMC12691371; doi:10.3390/cancers17233876)
Supplement: Supplementary file 1 [file cancers-17-03876-s001.zip › Table S1.pdf]

Supply Table S1. DEG gene list of Poor responder vs Good responder (Baseline)

Gene	LogFC	AveExpr	t	P.Value	Adj.P.Val	Ave_Pre_RE	Ave_Pre_SE
IP6K2	0.7070861	8.4958556	3.3994523	0.0014301	0.9796535	8.7369077	8.0298216
MGP	1.4494388	6.8265718	3.3898444	0.0014704	0.9796535	7.3206987	5.8712599
PRR34-AS1	0.6233094	8.2686373	3.1937746	0.0025735	0.9796535	8.4811291	7.8578197
RND3	0.5607310	9.9516268	3.0706138	0.0036270	0.9796535	10.1427851	9.5820541
LOC100507316	0.6191053	5.7268347	2.9717541	0.0047533	0.9796535	5.9378933	5.3187880
VWCE	0.5938945	6.0662817	2.9066129	0.0056661	0.9796535	6.2687458	5.6748512
RRAD	0.5487298	4.8522190	2.9063060	0.0056707	0.9796535	5.0392860	4.4905562
KLHDC9	0.5764789	6.7630583	2.8989716	0.0057833	0.9796535	6.9595852	6.3831063
ECHDC2	0.5733214	7.8273363	2.8868760	0.0059734	0.9796535	8.0227868	7.4494654
ALS2CL	0.5924546	6.1425748	2.8499472	0.0065905	0.9796535	6.3445480	5.7520934
LYPD6B	1.0330318	6.3555563	2.8385594	0.0067924	0.9796535	6.7077262	5.6746944
WLS	1.0433853	8.1359282	2.8205445	0.0071236	0.9796535	8.4916277	7.4482425
CDH3	1.2012003	8.9082108	2.8027152	0.0074661	0.9796535	9.3177109	8.1165106
NEK10	0.5873777	5.1436688	2.7871860	0.0077768	0.9796535	5.3439112	4.7565335
GNG12	0.6619253	6.1006774	2.7590200	0.0083711	0.9796535	6.3263338	5.6644085
LOC102724587	0.6255023	4.0929116	2.6957845	0.0098613	0.9796535	4.3061511	3.6806488

Gene	LogFC	AveExpr	t	P.Value	Adj.P.Val	Ave_Pre_RE	Ave_Pre_SE
WFDC2	1.1610925	7.6386314	2.6913752	0.0099738	0.9796535	8.0344584	6.8733660
HYI	0.5735311	7.3279409	2.6800860	0.0102673	0.9796535	7.5234628	6.9499318
LOC642776	0.6422329	4.9616045	2.6785397	0.0103081	0.9796535	5.1805476	4.5383147
CP	1.6754903	6.9166368	2.6767043	0.0103567	0.9796535	7.4878267	5.8123364
ABLIM1	0.5915778	7.5432535	2.6668677	0.0106210	0.9796535	7.7449278	7.1533500
SPG20	0.5988436	5.2192161	2.6444022	0.0112480	0.9796535	5.4233673	4.8245237
WDR78	0.6591971	4.9075425	2.6404058	0.0113631	0.9796535	5.1322688	4.4730717
LOC340184	0.6178051	3.3390864	2.6346646	0.0115302	0.9796535	3.5497018	2.9318967
ATHL1	0.8096685	8.2697809	2.5943223	0.0127698	0.9796535	8.5458042	7.7361357
KIF9	0.5810945	8.6949677	2.5871828	0.0130015	0.9796535	8.8930680	8.3119736
PCLO	0.8870501	5.5428645	2.5673123	0.0136666	0.9796535	5.8452680	4.9582178
F3	0.7758498	7.9447295	2.5568272	0.0140300	0.9796535	8.2092238	7.4333739
ANXA1	0.9055023	4.9607904	2.5368967	0.0147451	0.9796535	5.2694844	4.3639821
LINC00472	0.7825345	5.9821594	2.5268665	0.0151174	0.9796535	6.2489325	5.4663980
ATP1B1	0.8783084	8.9465676	2.5040997	0.0159945	0.9796535	9.2459909	8.3676826
PON3	1.0269922	6.9354782	2.4619505	0.0177411	0.9796535	7.2855892	6.2585970
SERTAD4-AS1	0.5597763	6.4182238	2.4406547	0.0186877	0.9796535	6.6090567	6.0492803

Gene	LogFC	AveExpr	t	P.Value	Adj.P.Val	Ave_Pre_RE	Ave_Pre_SE
DHCR24	0.8606072	9.7872669	2.4357268	0.0189132	0.9796535	10.0806558	9.2200486
NFIA	0.8872818	6.1898340	2.4165341	0.0198149	0.9796535	6.4923164	5.6050346
HOXC9	0.5714560	7.0632602	2.3934072	0.0209529	0.9796535	7.2580747	6.6866187
ADM	0.8034851	9.7815263	2.3862363	0.0213176	0.9796535	10.0554417	9.2519566
WIF1	1.5549331	6.1173205	2.3790273	0.0216899	0.9796535	6.6474113	5.0924782
NPR3	1.1314060	6.0087944	2.3758094	0.0218580	0.9796535	6.3945010	5.2630950
CARD10	0.6188017	6.8882770	2.3518020	0.0231493	0.9796535	7.0992321	6.4804304
THRB	0.8494088	6.8136500	2.2905219	0.0267612	0.9796535	7.1032212	6.2538123
ANGPTL4	0.7235870	7.3428546	2.2862356	0.0270317	0.9796535	7.5895320	6.8659450
SNCAIP	0.7742900	6.9278200	2.2700998	0.0280722	0.9796535	7.1917825	6.4174925
CMBL	0.9253865	8.2913208	2.2660357	0.0283398	0.9796535	8.6067935	7.6814070
NTRK2	1.3251069	8.0097067	2.2656483	0.0283654	0.9796535	8.4614476	7.1363408
SFN	0.7842141	6.8106048	2.2514007	0.0293224	0.9796535	7.0779505	6.2937364
LAMB3	0.8681786	8.1337295	2.2470765	0.0296185	0.9796535	8.4296994	7.5615209
VWDE	0.7910405	4.8359356	2.2328791	0.0306093	0.9796535	5.1056085	4.3145679
SMARCD3	0.5673877	7.5985316	2.2272958	0.0310070	0.9796535	7.7919593	7.2245716
SART1	0.8570162	6.1484066	2.2011266	0.0329326	0.9796535	6.4405712	5.5835550

Gene	LogFC	AveExpr	t	P.Value	Adj.P.Val	Ave_Pre_RE	Ave_Pre_SE
BCL6	0.5673257	7.2866547	2.1987383	0.0331135	0.9796535	7.4800612	6.9127355
TNFSF15	0.7612942	6.8511639	2.1870293	0.0340132	0.9796535	7.1106960	6.3494018
LURAP1L	0.7071640	7.2116040	2.1825720	0.0343613	0.9796535	7.4526826	6.7455186
SOCS2-AS1	0.5637787	4.3038576	2.1536297	0.0366992	0.9796535	4.4960549	3.9322761
PEG3-AS1	0.9715279	5.7751514	2.1506978	0.0369436	0.9796535	6.1063541	5.1348262
LAMC2	0.7414919	7.1252664	2.1486718	0.0371134	0.9796535	7.3780477	6.6365558
AGT	0.7149830	6.2339074	2.1445781	0.0374585	0.9796535	6.4776516	5.7626686
CNTNAP3P2	0.6329079	4.2281661	2.1367133	0.0381295	0.9796535	4.4439302	3.8110222
PCOLCE2	1.3567825	7.6757070	2.1318690	0.0385481	0.9796535	8.1382465	6.7814640
ZNF204P	0.5421190	5.8183196	2.1152850	0.0400116	0.9796535	6.0031329	5.4610139
PDK4	1.2342680	8.8113036	2.0987339	0.0415206	0.9796535	9.2320768	7.9978087
KANK1	0.5969143	8.4029874	2.0955598	0.0418156	0.9796535	8.6064809	8.0095666
LOC100288911	0.6436549	6.9361644	2.0925179	0.0421001	0.9796535	7.1555922	6.5119373
CLCN4	0.5722029	6.5980009	2.0902095	0.0423171	0.9796535	6.7930700	6.2208671
ATP8B4	0.5876074	4.9236960	2.0893122	0.0424017	0.9796535	5.1240167	4.5364094
STAC2	0.6272538	7.1879453	2.0774067	0.0435383	0.9796535	7.4017818	6.7745280
MAP2	0.6752886	7.3380438	2.0742216	0.0438469	0.9796535	7.5682558	6.8929672

Gene	LogFC	AveExpr	t	P.Value	Adj.P.Val	Ave_Pre_RE	Ave_Pre_SE
PTPRM	0.5658418	9.0285513	2.0655799	0.0446937	0.9796535	9.2214520	8.6556101
SBSPON	0.8821889	6.4470565	2.0542547	0.0458251	0.9796535	6.7478027	5.8656138
TLCD2	0.6782804	5.4675577	2.0434298	0.0469297	0.9796535	5.6987897	5.0205093
CLU	0.8263132	7.5866230	2.0200400	0.0493958	0.9796535	7.8683207	7.0420075
CMYA5	0.7053186	6.9386171	2.0183707	0.0495761	0.9796535	7.1790666	6.4737481
**Comparison_Group: Baseline/Post-chemotherapy**							
Gene	logFC	AveExpr	t	P.Value	Adj.P.Val	Ave_Post_RE	Ave_Post_SE
ZBTB8OS	0.8638462	8.2484872	5.4595564	0.0000582	0.1204531	8.4788462	7.615
ARMC9	0.9292308	6.0064359	5.3269939	0.0000752	0.1204531	6.2542308	5.325
NKAP	0.7676923	7.3304744	5.3053448	0.0000784	0.1204531	7.5351923	6.768
ABCC1	1.0692308	5.9366026	5.2213839	0.0000923	0.1229496	6.2217308	5.153
XPO4	1.0138462	5.8609872	5.1732169	0.0001015	0.1264266	6.1313462	5.118
PAPSS2	1.3230769	9.4427564	5.1432471	0.0001076	0.1264266	9.7955769	8.473
MRPL57	0.8669231	6.9632436	5.0861988	0.0001204	0.1284143	7.1944231	6.328
HGSNAT	0.7053846	4.1572821	5.0787851	0.0001222	0.1284143	4.3453846	3.640
PGM2	0.8715385	9.7391282	4.9092446	0.0001709	0.1578532	9.9715385	9.100
RBM19	0.7142308	6.5462692	4.8883803	0.0001782	0.1578532	6.7367308	6.023

Gene	logFC	AveExpr	t	P.Value	Adj.P.Val	Ave_Post_RE	Ave_Post_SE
GAS2L3	1.8019231	8.0539103	4.8498797	0.0001924	0.1578532	8.5344231	6.733
BRCA1	1.3019231	6.9997436	4.8169963	0.0002055	0.1578532	7.3469231	6.045
FGFR1OP	1.2353846	7.0884487	4.7961509	0.0002142	0.1584864	7.4178846	6.183
RGS1	1.4111538	9.9648462	4.6160437	0.0003079	0.1863428	10.3411538	8.930
RCOR2	0.6684615	6.4502051	4.4978574	0.0003913	0.2206577	6.6284615	5.960
NDC1	1.4319231	7.0700769	4.4699573	0.0004141	0.2206577	7.4519231	6.020
TTC39C	0.9969231	6.4110769	4.3448832	0.0005346	0.2280804	6.6769231	5.680
KCNH2	0.7803846	6.2147821	4.1935527	0.0007294	0.2421086	6.4228846	5.643
B4GALNT1	1.2246154	5.8930513	4.1721457	0.0007623	0.2421086	6.2196154	4.995
TNFAIP6	1.6138462	7.6509872	4.1453343	0.0008056	0.2421086	8.0813462	6.468
EP400NL	1.2084615	6.1962051	4.1419356	0.0008113	0.2421086	6.5184615	5.310
MKI67	2.1384615	6.6007051	4.1411005	0.0008127	0.2421086	7.1709615	5.033
MRPS16	0.7153846	8.3021154	4.1341027	0.0008245	0.2421086	8.4928846	7.778
ATP11A	1.1311538	8.5995128	4.1243800	0.0008412	0.2421086	8.9011538	7.770
LEPROTL1	0.9234615	8.5197051	4.0952762	0.0008933	0.2421086	8.7659615	7.843
ARL6IP1	0.7230769	11.5027564	4.0886754	0.0009056	0.2421086	11.6955769	10.973
CNDP2	0.6234615	10.3372051	4.0886091	0.0009057	0.2421086	10.5034615	9.880

Gene	logFC	AveExpr	t	P.Value	Adj.P.Val	Ave_Post_RE	Ave_Post_SE
SLC7A6	0.9642308	6.2171026	4.0648466	0.0009513	0.2421086	6.4742308	5.510
WISP1	2.4784615	7.6575385	4.0638821	0.0009532	0.2421086	8.3184615	5.840
FAM107B	0.7684615	9.3110385	4.0508329	0.0009793	0.2421086	9.5159615	8.748
CXCR4	1.4126923	11.3309744	4.0495784	0.0009818	0.2421086	11.7076923	10.295
APOOL	1.0703846	5.9924487	4.0341154	0.0010137	0.2469304	6.2778846	5.208
GPD2	0.8619231	5.6795769	3.9913813	0.0011075	0.2525995	5.9094231	5.048
ETV2	0.5930769	4.9324231	3.9840355	0.0011245	0.2525995	5.0905769	4.498
TXNL4A	0.9353846	10.5984487	3.9436765	0.0012226	0.2605005	10.8478846	9.913
LPCAT1	0.7530769	8.9097564	3.9423712	0.0012259	0.2605005	9.1105769	8.358
UNC5B	1.4388462	7.3776538	3.9335993	0.0012485	0.2612166	7.7613462	6.323
MTDH	0.7273077	10.1833590	3.8879439	0.0013726	0.2762882	10.3773077	9.650
SRPK1	0.9065385	6.5122949	3.8575220	0.0014621	0.2839846	6.7540385	5.848
RC3H2	0.7969231	5.9994103	3.8571697	0.0014632	0.2839846	6.2119231	5.415
KIAA1211	1.8576923	5.7098077	3.8486386	0.0014893	0.2860379	6.2051923	4.348
TENM4	0.7069231	5.7534103	3.8060669	0.0016272	0.2923538	5.9419231	5.235
KAZALD1	0.5546154	6.1817179	3.7975556	0.0016562	0.2923538	6.3296154	5.775
DFFA	0.6030769	7.1022564	3.7954101	0.0016636	0.2923538	7.2630769	6.660

Gene	logFC	AveExpr	t	P.Value	Adj.P.Val	Ave_Post_RE	Ave_Post_SE
GAL	1.8842308	6.3642692	3.7874996	0.0016912	0.2923538	6.8667308	4.983
SNX5	0.8892308	6.2071026	3.7582858	0.0017972	0.3051476	6.4442308	5.555
PAFAH1B1	1.1965385	8.7999615	3.7481477	0.0018356	0.3051476	9.1190385	7.923
RCL1	0.7803846	7.4872821	3.7479730	0.0018362	0.3051476	7.6953846	6.915
FAM155A	1.3207692	4.6535641	3.7422456	0.0018583	0.3051476	5.0057692	3.685
MRPL36	0.7730769	10.0544231	3.7308845	0.0019027	0.3089860	10.2605769	9.488
WFDC1	0.7746154	6.8155513	3.7142072	0.0019700	0.3136517	7.0221154	6.248
C4orf48	1.2134615	8.3948718	3.7121148	0.0019786	0.3136517	8.7184615	7.505
LOC101929504	0.9219231	5.1485769	3.6986017	0.0020351	0.3169207	5.3944231	4.473
COQ2	0.8926923	8.2096410	3.6814607	0.0021090	0.3169207	8.4476923	7.555
ETF1	0.5853846	9.4517821	3.6773168	0.0021273	0.3169207	9.6078846	9.023
LARS2	0.7092308	7.5726026	3.6755680	0.0021351	0.3169207	7.7617308	7.053
NDUFS6	0.7692308	11.2391026	3.6742672	0.0021409	0.3169207	11.4442308	10.675
GTF2H3	0.6292308	7.2864359	3.6713769	0.0021538	0.3169207	7.4542308	6.825
HOXD13	0.5700000	5.1530000	3.6526883	0.0022393	0.3221550	5.3050000	4.735
UBE2K	0.7196154	9.7627179	3.6412507	0.0022933	0.3221550	9.9546154	9.235
BGN	1.6488462	9.4841538	3.6337362	0.0023295	0.3221550	9.9238462	8.275

Gene	logFC	AveExpr	t	P.Value	Adj.P.Val	Ave_Post_RE	Ave_Post_SE
RRP36	1.1469231	8.7885769	3.6334103	0.0023311	0.3221550	9.0944231	7.948
GTF2IRD2	0.8661538	8.2351795	3.6306422	0.0023446	0.3221550	8.4661538	7.600
EME1	1.0396154	7.0073846	3.6162071	0.0024162	0.3221550	7.2846154	6.245
TRAPPC5	0.6361538	8.6915128	3.6026858	0.0024853	0.3221550	8.8611538	8.225
COL10A1	3.4588462	7.7989872	3.5919602	0.0025415	0.3221550	8.7213462	5.263
KIFC2	0.9819231	6.5275769	3.5775473	0.0026190	0.3221550	6.7894231	5.808
ASCC1	0.7311538	7.6761795	3.5700990	0.0026600	0.3221550	7.8711538	7.140
MBD5	0.6500000	6.2766667	3.5594083	0.0027199	0.3221550	6.4500000	5.800
GAPVD1	0.8761538	7.5775128	3.5591890	0.0027212	0.3221550	7.8111538	6.935
XXYLT1	0.7311538	6.7411795	3.5583900	0.0027257	0.3221550	6.9361538	6.205
LINC00922	0.6950000	5.0221667	3.5528994	0.0027571	0.3221550	5.2075000	4.513
DCK	0.7069231	8.3734103	3.5527365	0.0027580	0.3221550	8.5619231	7.855
R3HDM1	0.7673077	9.0951923	3.5480189	0.0027853	0.3228251	9.2998077	8.533
VKORC1	0.5884615	9.9615385	3.5341184	0.0028672	0.3236352	10.1184615	9.530
KPNA2	1.2700000	10.9138333	3.5316514	0.0028820	0.3236352	11.2525000	9.983
APLF	0.7896154	4.0365513	3.5278466	0.0029049	0.3236352	4.2471154	3.458
TMEM57	0.6803846	7.1389487	3.5213714	0.0029444	0.3236352	7.3203846	6.640

Gene	logFC	AveExpr	t	P.Value	Adj.P.Val	Ave_Post_RE	Ave_Post_SE
COMP	2.6300000	7.6261667	3.5058086	0.0030415	0.3283810	8.3275000	5.698
ADA	1.0392308	7.6071026	3.4908781	0.0031376	0.3351100	7.8842308	6.845
TBC1D16	0.9688462	7.4779872	3.4883624	0.0031541	0.3351100	7.7363462	6.768
SLC41A2	0.8438462	6.4588205	3.4376692	0.0035057	0.3572569	6.6838462	5.840
C11orf24	0.6500000	7.4991667	3.4286269	0.0035724	0.3584234	7.6725000	7.023
LOC389641	0.5830769	5.4350897	3.4264116	0.0035889	0.3584234	5.5905769	5.008
NACC1	0.9373077	7.4498590	3.3798400	0.0039546	0.3608956	7.6998077	6.763
THEM4	0.6515385	5.7427949	3.3764683	0.0039825	0.3608956	5.9165385	5.265
ATL3	0.9161538	7.4393462	3.3732925	0.0040089	0.3608956	7.6836538	6.768
PLK1	1.4053846	6.1981154	3.3729025	0.0040122	0.3608956	6.5728846	5.168
TMEM45A	1.8476923	9.2699744	3.3701309	0.0040354	0.3608956	9.7626923	7.915
PDE10A	1.0300000	6.3203333	3.3674861	0.0040577	0.3608956	6.5950000	5.565
CHST8	0.6526923	6.1611410	3.3501086	0.0042073	0.3608956	6.3351923	5.683
HIST1H1E	0.7103846	5.1359487	3.3485948	0.0042206	0.3608956	5.3253846	4.615
DYRK4	0.6450000	8.0830000	3.3454591	0.0042482	0.3608956	8.2550000	7.610
MCUR1	0.8869231	9.3929103	3.3252470	0.0044308	0.3652792	9.6294231	8.743
CCDC149	0.6107692	7.2928974	3.3176700	0.0045012	0.3654027	7.4557692	6.845

Gene	logFC	AveExpr	t	P.Value	Adj.P.Val	Ave_Post_RE	Ave_Post_SE
LAMTOR2	0.7773077	8.7450256	3.3139961	0.0045358	0.3654027	8.9523077	8.175
ATP11C	0.8269231	4.2239103	3.3103536	0.0045703	0.3654027	4.4444231	3.618
KMO	1.6950000	7.0230000	3.3081041	0.0045918	0.3654027	7.4750000	5.780
GPR4	0.7926923	6.3538077	3.2962880	0.0047061	0.3700764	6.5651923	5.773
OLA1	0.5546154	10.7467179	3.2933676	0.0047348	0.3708721	10.8946154	10.340
TDP1	0.7400000	7.9001667	3.2831071	0.0048369	0.3714100	8.0975000	7.358
GCN1L1	0.7111538	8.6490128	3.2803239	0.0048650	0.3714100	8.8386538	8.128
CORIN	1.0500000	5.2125000	3.2796968	0.0048714	0.3714100	5.4925000	4.443
TMEM38B	0.7526923	7.0719744	3.2763439	0.0049055	0.3714100	7.2726923	6.520
TLCD1	1.3242308	7.3286026	3.2741821	0.0049276	0.3714100	7.6817308	6.358
SFT2D2	1.0661538	7.7168462	3.2571404	0.0051054	0.3762905	8.0011538	6.935
HOXB9	0.7738462	6.1124872	3.2479780	0.0052036	0.3789188	6.3188462	5.545
PCCB	0.9326923	8.7814744	3.2456226	0.0052291	0.3789188	9.0301923	8.098
UBXN2B	0.8353846	6.5951154	3.2426534	0.0052615	0.3793972	6.8178846	5.983
AGTPBP1	0.8350000	8.1048333	3.2362451	0.0053320	0.3831020	8.3275000	7.493
HIF1A	0.6161538	11.8843462	3.2310365	0.0053901	0.3858840	12.0486538	11.433
ZRANB3	0.6953846	6.4124487	3.2201695	0.0055132	0.3898372	6.5978846	5.903

Gene	logFC	AveExpr	t	P.Value	Adj.P.Val	Ave_Post_RE	Ave_Post_SE
LRP8	1.9100000	6.4081667	3.2063639	0.0056736	0.3900855	6.9175000	5.008
WDR36	0.7176923	5.4013077	3.2013038	0.0057336	0.3900855	5.5926923	4.875
COL5A2	1.3273077	9.6933590	3.2009577	0.0057377	0.3900855	10.0473077	8.720
RNASEH2C	0.5738462	9.4458205	3.1999906	0.0057492	0.3900855	9.5988462	9.025
BCL9	0.7488462	6.9666538	3.1984548	0.0057676	0.3900855	7.1663462	6.418
ZNF354A	0.7030769	7.5880897	3.1976943	0.0057767	0.3900855	7.7755769	7.073
CDC123	0.8169231	10.0315769	3.1946031	0.0058139	0.3900855	10.2494231	9.433
LOXL1	0.9392308	8.1862692	3.1936520	0.0058254	0.3900855	8.4367308	7.498
GJB2	3.1115385	7.2892949	3.1889901	0.0058821	0.3900855	8.1190385	5.008
MIPEPP3	0.9800000	5.5436667	3.1815589	0.0059735	0.3931984	5.8050000	4.825
NMU	1.9034615	6.6183718	3.1701135	0.0061171	0.3931984	7.1259615	5.223
CHST1	0.7550000	6.0161667	3.1683237	0.0061399	0.3931984	6.2175000	5.463
SHFM1	0.6961538	10.6430128	3.1677523	0.0061472	0.3931984	10.8286538	10.133
FAM173A	0.8176923	7.7746410	3.1668513	0.0061587	0.3931984	7.9926923	7.175
SLC16A1	0.6338462	5.0398205	3.1623831	0.0062161	0.3931984	5.2088462	4.575
GNA12	0.5630769	7.5079231	3.1617132	0.0062247	0.3931984	7.6580769	7.095
CPT1A	0.9850000	4.6198333	3.1609601	0.0062344	0.3931984	4.8825000	3.898

Gene	logFC	AveExpr	t	P.Value	Adj.P.Val	Ave_Post_RE	Ave_Post_SE
ODF2	0.8326923	6.3381410	3.1558904	0.0063004	0.3931984	6.5601923	5.728
ASPHD2	0.8550000	6.3670000	3.1460785	0.0064299	0.3931984	6.5950000	5.740
TXNRD1	1.0319231	10.3492436	3.1445354	0.0064505	0.3931984	10.6244231	9.593
POC1A	1.1076923	6.9423077	3.1427188	0.0064749	0.3931984	7.2376923	6.130
VOPP1	0.6984615	10.4147051	3.1405922	0.0065035	0.3931984	10.6009615	9.903
GALE	0.8657692	7.2023974	3.1399523	0.0065121	0.3931984	7.4332692	6.568
CD109	0.6300000	5.5020000	3.1395201	0.0065179	0.3931984	5.6700000	5.040
NHLRC3	0.5669231	5.6632436	3.1394342	0.0065191	0.3931984	5.8144231	5.248
TAF2	0.9669231	9.4190769	3.1390825	0.0065239	0.3931984	9.6769231	8.710
C1orf233	0.8103846	7.0967821	3.1389089	0.0065262	0.3931984	7.3128846	6.503
NSUN2	0.7500000	9.1950000	3.1381087	0.0065370	0.3931984	9.3950000	8.645
BOLA2	0.8784615	4.1217051	3.1376373	0.0065434	0.3931984	4.3559615	3.478
UXS1	0.7673077	9.3276923	3.1376351	0.0065435	0.3931984	9.5323077	8.765
QPRT	1.2546154	7.1675513	3.1295102	0.0066546	0.3931984	7.5021154	6.248
USP45	1.0507692	4.9180641	3.1267890	0.0066923	0.3931984	5.1982692	4.148
ZDHHC20	0.6600000	4.6740000	3.1259927	0.0067033	0.3931984	4.8500000	4.190
PNKD	1.1996154	7.4347179	3.1224194	0.0067532	0.3931984	7.7546154	6.555

Gene	logFC	AveExpr	t	P.Value	Adj.P.Val	Ave_Post_RE	Ave_Post_SE
SLC12A8	1.3119231	7.2620769	3.1223695	0.0067539	0.3931984	7.6119231	6.300
SLC7A11	1.7646154	5.2840513	3.1208718	0.0067748	0.3931984	5.7546154	3.990
SLC29A2	0.9946154	6.1693846	3.1177383	0.0068190	0.3931984	6.4346154	5.440
GTF2E2	0.5400000	8.7910000	3.1168013	0.0068322	0.3931984	8.9350000	8.395
UBE3C	0.5769231	5.2955769	3.1161241	0.0068418	0.3931984	5.4494231	4.873
TPRXL	0.7215385	5.5591282	3.1137552	0.0068755	0.3931984	5.7515385	5.030
UBFD1	0.9400000	7.2443333	3.1077639	0.0069614	0.3950183	7.4950000	6.555
ADAMTS6	1.2184615	5.7560385	3.0942544	0.0071589	0.4016625	6.0809615	4.863
KNTC1	1.1423077	7.0176923	3.0918294	0.0071949	0.4019058	7.3223077	6.180
ANP32E	1.7930769	8.3124231	3.0912563	0.0072035	0.4019058	8.7905769	6.998
SNORD89	0.5500000	7.1158333	3.0818723	0.0073448	0.4047132	7.2625000	6.713
DROSHA	0.5530769	8.9455897	3.0761912	0.0074316	0.4047132	9.0930769	8.540
KLHL7	1.2361538	7.1190128	3.0693473	0.0075376	0.4047132	7.4486538	6.213
TXNDC9	0.5776923	5.5236410	3.0678580	0.0075609	0.4047132	5.6776923	5.100
SAR1A	0.7130769	6.6179231	3.0668268	0.0075770	0.4047132	6.8080769	6.095
FCRLB	0.7946154	6.4227179	3.0644823	0.0076139	0.4047132	6.6346154	5.840
NUSAP1	1.5380769	6.2779231	3.0625897	0.0076437	0.4047132	6.6880769	5.150

Gene	logFC	AveExpr	t	P.Value	Adj.P.Val	Ave_Post_RE	Ave_Post_SE
PTTG1	1.7711538	9.7588462	3.0590722	0.0076996	0.4047132	10.2311538	8.460
FXN	0.7796154	5.9542179	3.0546215	0.0077708	0.4055585	6.1621154	5.383
OLFML2B	1.1673077	8.1310256	3.0478315	0.0078806	0.4060131	8.4423077	7.275
WSB2	0.7080769	10.1667564	3.0467966	0.0078975	0.4060131	10.3555769	9.648
SLC52A2	1.3253846	7.4969487	3.0443938	0.0079368	0.4060131	7.8503846	6.525
COPB1	0.5511538	11.6116795	3.0387948	0.0080292	0.4060131	11.7586538	11.208
ARPC3	0.6026923	10.8869744	3.0365865	0.0080659	0.4068402	11.0476923	10.445
ARSI	0.8146154	5.9248846	3.0331076	0.0081241	0.4087431	6.1421154	5.328
PPEF1	0.8734615	4.5505385	3.0282722	0.0082057	0.4100036	4.7834615	3.910
LDHA	0.5634615	12.8107051	3.0250534	0.0082604	0.4105028	12.9609615	12.398
NDUFV3	0.7473077	9.3630256	3.0249695	0.0082618	0.4105028	9.5623077	8.815
SLC45A4	0.8761538	7.9725128	3.0221301	0.0083104	0.4108731	8.2061538	7.330
AGPAT5	0.8996154	9.2672179	3.0102417	0.0085170	0.4145558	9.5071154	8.608
DDX39A	0.9407692	9.5498974	3.0099175	0.0085227	0.4145558	9.8007692	8.860
FLVCR1	0.9780769	9.2597564	3.0091617	0.0085360	0.4145558	9.5205769	8.543
TNFAIP8L1	0.7030769	7.8930897	3.0078575	0.0085590	0.4145558	8.0805769	7.378
ZNF706	0.7684615	11.1385385	3.0059651	0.0085925	0.4145558	11.3434615	10.575

Gene	logFC	AveExpr	t	P.Value	Adj.P.Val	Ave_Post_RE	Ave_Post_SE
IMMT	0.6842308	9.8017692	3.0035675	0.0086351	0.4156083	9.9842308	9.300
CCNB2	2.4807692	8.4642308	2.9979738	0.0087353	0.4182090	9.1257692	6.645
SBSN	0.6269231	4.7947436	2.9929491	0.0088264	0.4207586	4.9619231	4.335
FANCI	0.9965385	5.6782949	2.9908413	0.0088648	0.4215217	5.9440385	4.948
HHIPL1	0.6842308	5.0442692	2.9875360	0.0089255	0.4215217	5.2267308	4.543
RINT1	0.5665385	7.9379615	2.9874573	0.0089269	0.4215217	8.0890385	7.523
PIM1	0.5657692	7.9623974	2.9777089	0.0091082	0.4215217	8.1132692	7.548
VPREB3	0.8042308	5.4122692	2.9733740	0.0091899	0.4215217	5.6267308	4.823
SGPP2	1.0107692	5.5212308	2.9726432	0.0092038	0.4215217	5.7907692	4.780
SLC2A6	0.8469231	6.0385769	2.9720299	0.0092154	0.4215217	6.2644231	5.418
RABIF	0.7850000	8.5331667	2.9708570	0.0092377	0.4215217	8.7425000	7.958
RRP15	0.9180769	8.6307564	2.9697587	0.0092586	0.4215217	8.8755769	7.958
ACBD6	0.8642308	8.3712692	2.9665849	0.0093194	0.4215217	8.6017308	7.738
NUF2	2.6953846	6.8791154	2.9664587	0.0093218	0.4215217	7.5978846	4.903
CPEB2	0.6023077	6.0841923	2.9629120	0.0093902	0.4215217	6.2448077	5.643
GPR183	1.3161538	6.8101795	2.9586786	0.0094724	0.4215217	7.1611538	5.845
ARL1	0.5784615	9.2867051	2.9583195	0.0094794	0.4215217	9.4409615	8.863

Gene	logFC	AveExpr	t	P.Value	Adj.P.Val	Ave_Post_RE	Ave_Post_SE
P4HA1	0.9569231	9.3867436	2.9554012	0.0095366	0.4215217	9.6419231	8.685
C18orf8	0.6438462	8.1871538	2.9492388	0.0096584	0.4215217	8.3588462	7.715
CTHRC1	1.2519231	11.4580769	2.9492153	0.0096588	0.4215217	11.7919231	10.540
HEATR3	0.8557692	5.4625641	2.9462812	0.0097173	0.4215217	5.6907692	4.835
SLC25A32	0.9626923	9.1309744	2.9441870	0.0097593	0.4215217	9.3876923	8.425
RAB10	0.6553846	9.4881154	2.9404340	0.0098350	0.4215217	9.6628846	9.008
LEPREL4	1.1653846	7.5146154	2.9396138	0.0098516	0.4215217	7.8253846	6.660
TMEM167A	0.8361538	9.4981795	2.9377195	0.0098901	0.4215217	9.7211538	8.885
HEYL	1.1834615	6.7103718	2.9356613	0.0099321	0.4215217	7.0259615	5.843
HAS2	0.9034615	5.8125385	2.9326768	0.0099932	0.4215217	6.0534615	5.150
DPY19L1	0.7050000	6.0670000	2.9324314	0.0099983	0.4215217	6.2550000	5.550
