# Supplementary material for: CRISPR-Cas9 Genome and Double-Knockout Screening to Identify Novel Therapeutic Targets for Chemoresistance in Triple-Negative Breast Cancer
Source: Cancers (Basel). 2025 Dec 3;17(23):3876. doi: 10.3390/cancers17233876 (PMC12691371; doi:10.3390/cancers17233876)
Supplement: Supplementary file 1 [file cancers-17-03876-s001.zip › Table S2.pdf]

Supply Table S2. TNBC cell line correlation score with poor responder (Baseline)

Name	Rank	Median.correlation
HCC70	5	0.4203756
HCC1187	6	0.4193687
HCC38	13	0.4107166
HCC1395	16	0.4101676
HCC1143	17	0.4083153
HCC2157	28	0.4025514
MDA-MB-468	38	0.3955186
HCC1937	56	0.3866011
MDA-MB-157	72	0.3811564
MDA-MB-231	92	0.3695660
CAL-85-1	94	0.3692342
BT-20	107	0.3647778
Hs 578T	124	0.3587171
HCC1806	157	0.3506537
HCC1599	168	0.3481556
BT-549	172	0.3471473
MDA-MB-436	250	0.3287118
CAL-148	349	0.3119059
CAL-51	355	0.3113053
CAL-120	383	0.3062729
DU4475	729	0.2357261
