# Supplementary material for: CRISPR-Cas9 Genome and Double-Knockout Screening to Identify Novel Therapeutic Targets for Chemoresistance in Triple-Negative Breast Cancer
Source: Cancers (Basel). 2025 Dec 3;17(23):3876. doi: 10.3390/cancers17233876 (PMC12691371; doi:10.3390/cancers17233876)
Supplement: Supplementary file 1 [file cancers-17-03876-s001.zip › Table S3.pdf]

Supply Table S3. TNBC cell line correlation score with poor responder (Post Chemo)

Name	Rank	Median.correlation
HCC1143	17	0.423177659
HCC38	20	0.421329397
HCC1395	26	0.415119249
HCC1187	38	0.408504434
MDA-MB-157	43	0.401938052
MDA-MB-231	69	0.389617663
HCC70	70	0.389128401
MDA-MB-468	85	0.380524322
Hs 578T	93	0.377051453
HCC2157	128	0.362521328
BT-549	134	0.359981553
MDA-MB-436	139	0.358436208
HCC1937	156	0.354023328
CAL-85-1	173	0.349118192
CAL-51	195	0.342772941
HCC1806	233	0.33445646
BT-20	238	0.33191512
HCC1599	240	0.33128585
CAL-120	258	0.327141755
CAL-148	421	0.291930731
DU4475	796	0.211459449
